# p38α‐MAPK‐deficient myeloid cells ameliorate symptoms and pathology of APP‐transgenic Alzheimer's disease mice

**DOI:** 10.1111/acel.13679

**Published:** 2022-07-31

**Authors:** Qinghua Luo, Laura Schnöder, Wenlin Hao, Kathrin Litzenburger, Yann Decker, Inge Tomic, Michael D. Menger, Yang Liu, Klaus Fassbender

**Affiliations:** ^1^ Department of Neurology Saarland University Homburg Germany; ^2^ German Institute for Dementia Prevention (DIDP) Saarland University Homburg Germany; ^3^ Institute for Clinical and Experimental Surgery Saarland University Homburg Germany

**Keywords:** Alzheimer's disease, amyloid‐beta (Aβ), microglia, neurodegeneration, p38α‐MAPK

## Abstract

Alzheimer's disease (AD), the most common cause of dementia in the elderly, is pathologically characterized by extracellular deposition of amyloid‐β peptides (Aβ) and microglia‐dominated inflammatory activation in the brain. p38α‐MAPK is activated in both neurons and microglia. How p38α‐MAPK in microglia contributes to AD pathogenesis remains unclear. In this study, we conditionally knocked out p38α‐MAPK in all myeloid cells or specifically in microglia of APP‐transgenic mice, and examined animals for AD‐associated pathologies (i.e., cognitive deficits, Aβ pathology, and neuroinflammation) and individual microglia for their inflammatory activation and Aβ internalization at different disease stages (e.g., at 4 and 9 months of age). Our experiments showed that p38α‐MAPK‐deficient myeloid cells were more effective than p38α‐MAPK‐deficient microglia in reducing cerebral Aβ and neuronal impairment in APP‐transgenic mice. Deficiency of p38α‐MAPK in myeloid cells inhibited inflammatory activation of individual microglia at 4 months but enhanced it at 9 months. Inflammatory activation promoted microglial internalization of Aβ. Interestingly, p38α‐MAPK‐deficient myeloid cells reduced IL‐17a‐expressing CD4‐positive lymphocytes in 9 but not 4‐month‐old APP‐transgenic mice. By cross‐breeding APP‐transgenic mice with *Il‐17a*‐knockout mice, we observed that IL‐17a deficiency potentially activated microglia and reduced Aβ deposition in the brain as shown in 9‐month‐old myeloid p38α‐MAPK‐deficient AD mice. Thus, p38α‐MAPK deficiency in all myeloid cells, but not only in microglia, prevents AD progression. IL‐17a‐expressing lymphocytes may partially mediate the pathogenic role of p38α‐MAPK in peripheral myeloid cells. Our study supports p38α‐MAPK as a therapeutic target for AD patients.

## Graphical abstract

Deficiency of p38α‐MAPK in myeloid cells, but not only in microglia, efficiently attenuates cerebral Aβ and improves cognitive function in APP‐transgenic Alzheimer's disease mice. As possible mechanisms, p38α‐MAPK‐deficient myeloid cells decrease peripheral IL‐17a‐expressing CD4‐positive cells and increase inflammatory activation and Aβ internalization in microglia.

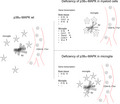

**Abbreviations**
AD: Alzheimer's disease
Aβ: amyloid‐β peptide
APP: amyloid precursor protein
APP(tg): APP‐transgenic
APP(wt): non‐APP‐transgenic
BMDMs: bone marrow‐derived macrophages
GFP: green fluorescence protein
IL‐17a: interleukin‐17a
LPS: lipopolysaccharide
NFT: neurofibrillary tangles
p‐tau: hyper‐phosphorylated tau protein
p38α‐MAPK: p38α mitogen‐activated protein kinase
RFP: red fluorescent protein
SR‐A: scavenger receptor A
Th17: T‐helper 17
TREM2: triggering receptor expressed on myeloid cells‐2

## 1 INTRODUCTION

Alzheimer's disease (AD), the major cause of dementia in the elderly, is pathologically characterized by three components: (i) extracellular deposits of amyloid‐β peptide (Aβ), (ii) intracellular neurofibrillary tangles (NFT) that is composed of hyper‐phosphorylated tau protein (p‐tau), and (iii) microglia‐dominated inflammatory activation in the brain parenchyma (Scheltens et al., 2021). Interactions between Aβ, p‐tau and inflammatory activation are primarily responsible for the progressive neurodegeneration in AD. However, many clinical trials to reduce Aβ accumulation or p‐tau aggregation or inflammatory activation (Pleen & Townley, 2022) have failed to produce AD therapies that modify the disease progression. A simple explanation for these failures is that the study population may have already reached a disease stage too late for effective intervention. However, it is important to recognize that AD is a heterogeneous disease. For example, the pathological and biochemical features of Aβ deposits or molecular structure of Aβ aggregates in the brain (Thal et al., 2015) varies among AD patients. Variations in Aβ structure affect how microglia respond to the Aβ deposits, which, in turn, affects inflammatory activation and Aβ internalization (Parvathy et al., 2009). A growing number of subtypes of activated microglia have recently been identified in AD brains (Chen & Colonna, 2021). Moreover, pathological examination of postmortem brain tissues and imaging studies show different distributions of tau‐related pathology and patterns of brain atrophy in AD patients (Ferreira et al., 2020). Therefore, targeting multiple pathogenic pathways might be more effective as a therapeutic intervention than focusing on a single step in AD disease progression.

p38α mitogen‐activated protein kinase (p38α‐MAPK) is a protein kinase present in a variety of cells that respond to external stress stimuli (Kumar et al., 2003). p38α‐MAPK is activated in both neurons and microglia in brains of AD patients (Hensley et al., 1999). Our recent study indicates that p38α‐MAPK deficiency in neurons reduces both Aβ and p‐tau levels in the brain of AD mice (Schnöder et al., 2016, 2020, 2021). A systemic administration of chemical p38α‐MAPK inhibitor has been observed to reduce inflammatory activation in the brain of APP‐ or tau‐transgenic mice (Bachstetter et al., 2012; Maphis et al., 2016). Thus, p38α‐MAPK inhibition might simultaneously target Aβ, p‐tau and inflammation in AD. A recent phase 2 clinical trial showed that a 24‐week treatment with p38α‐MAPK inhibitor decreased tau proteins in the cerebral spinal fluid of mild AD patients; although it did not improve the cognitive function (Prins et al., 2021). We believe that the therapeutic protocol can be optimized, if the pathogenic mechanisms of p38α‐MAPK are better understood. Pharmacological treatments with p38α‐MAPK inhibitors affect both microglial p38α‐MAPK and neuronal p38α‐MAPK, without the ability to distinguish their effects. The inhibition of inflammatory activation in the brain might come from neuronal p38α‐MAPK inhibition‐mediated attenuation of Aβ and p‐tau generation, or even from neuronal protection (Schnöder et al., 2020). In this study, we investigated specific effects of p38α‐MAPK in microglia or myeloid cells on AD pathogenesis.

The pathogenic role of microglia in AD is extremely heterogeneous. For example, the rare variants in the triggering receptor expressed on myeloid cells‐2 (TREM2) gene increase the risk of developing AD. One group reported that TREM2 deficiency in APP‐transgenic mice increases hippocampal Aβ burden and accelerates neuron loss (Wang et al., 2015); however, another group showed that TREM2 deletion reduces cerebral Aβ accumulation (Jay et al., 2015). Subsequent work suggested that the effect of TREM2 deficiency on cerebral Aβ accumulation depends on the stage of disease (Jay et al., 2017). Consistent with this conclusion is the observation from a longitudinal imaging study of human subjects with mild cognitive impairment that several peaks of microglial activation appear over the disease trajectory (Fan et al., 2017). These studies underscore the effects of the changing cellular environment and reinforce the idea that the pathogenic role of microglial activation should be dynamically investigated during disease progression.

In this study, we conditionally knocked out *Mapk14* gene (encoding p38α‐MAPK) in the myeloid cell lineage or specifically in microglia in amyloid precursor protein (APP)‐transgenic mice and investigated the AD pathology and microglial activation in early and late disease stages. We observed that deletion of p38α‐MAPK attenuated Aβ load and neuronal deficits of AD mice; however, the pathogenic mechanism of p38α‐MAPK is evolving during the disease progresses, which potentially involves peripheral interleukin (IL)‐17a‐expressing T lymphocytes.

## 2 RESULTS

### 2.1 Establishment of APP‐transgenic mice deficient of p38α‐MAPK in myeloid cells

To investigate the pathogenic role of p38α‐MAPK in microglia and peripheral myeloid cells in AD, we cross‐bred APP‐transgenic (APP^tg^) mice with p38α‐MAPK‐encoding gene *Mapk14* floxed mice, and LysM‐Cre^+/−^ mice expressing Cre specifically in the myeloid cell lineage, to obtain APP^tg^p38^fl/fl^LysM‐Cre^+/−^ (p38α deficient) and APP^tg^p38^fl/fl^LysM‐Cre^−/−^ (p38α wild type) of genotypes. By measuring *Mapk14* gene transcripts and p38‐MAPK proteins in CD11b^+^ brain cells from APP^tg^p38^fl/fl^LysM‐Cre^+/−^ and APP^tg^p38^fl/fl^LysM‐Cre^−/−^ mice, we found that the rate of LysM‐Cre‐mediated *Mapk14* gene recombination in microglia of 9‐month‐old AD mice was ~45% (Figure S1a,d,e). In 4‐month‐old AD mice, LysM‐Cre altered neither *Mapk14* transcription nor p38‐MAPK protein in CD11b+ brain cells, but decreased *Mapk14* transcription by 88% in CD11b+ blood cells (Figure S1b–e).

We further constructed APP‐transgenic green fluorescence protein (GFP)‐expressing LysM‐Cre reporter mice (APP^tg^ROSA^mT/mG^LysM‐Cre^+/−^; Muzumdar et al., 2007). GFP was mainly expressed in microglia associated with Aβ deposits (Figure S1f); Aβ deposits were also surrounded by microglia without expression of GFP, indicating heterogeneity of Aβ plaques. GFP was rarely expressed in neurons (Figure S1g). APP^tg^p38^fl/fl^LysM‐Cre^+/−^ mice were also mated to CCR2‐RFP reporter mice expressing red fluorescent protein (RFP) under the control of *Ccr2* gene promoter (Saederup et al., 2010). Both histological and flow cytometric analysis showed that p38α‐MAPK deficiency does not affect the recruitment of peripheral myeloid cells into the brain of 9‐month‐old APP‐transgenic mice (Figure S2).

### 2.2 Deficiency of p38α‐MAPK in myeloid cells improved the cognitive function of APP‐transgenic mice

**FIGURE 1:**
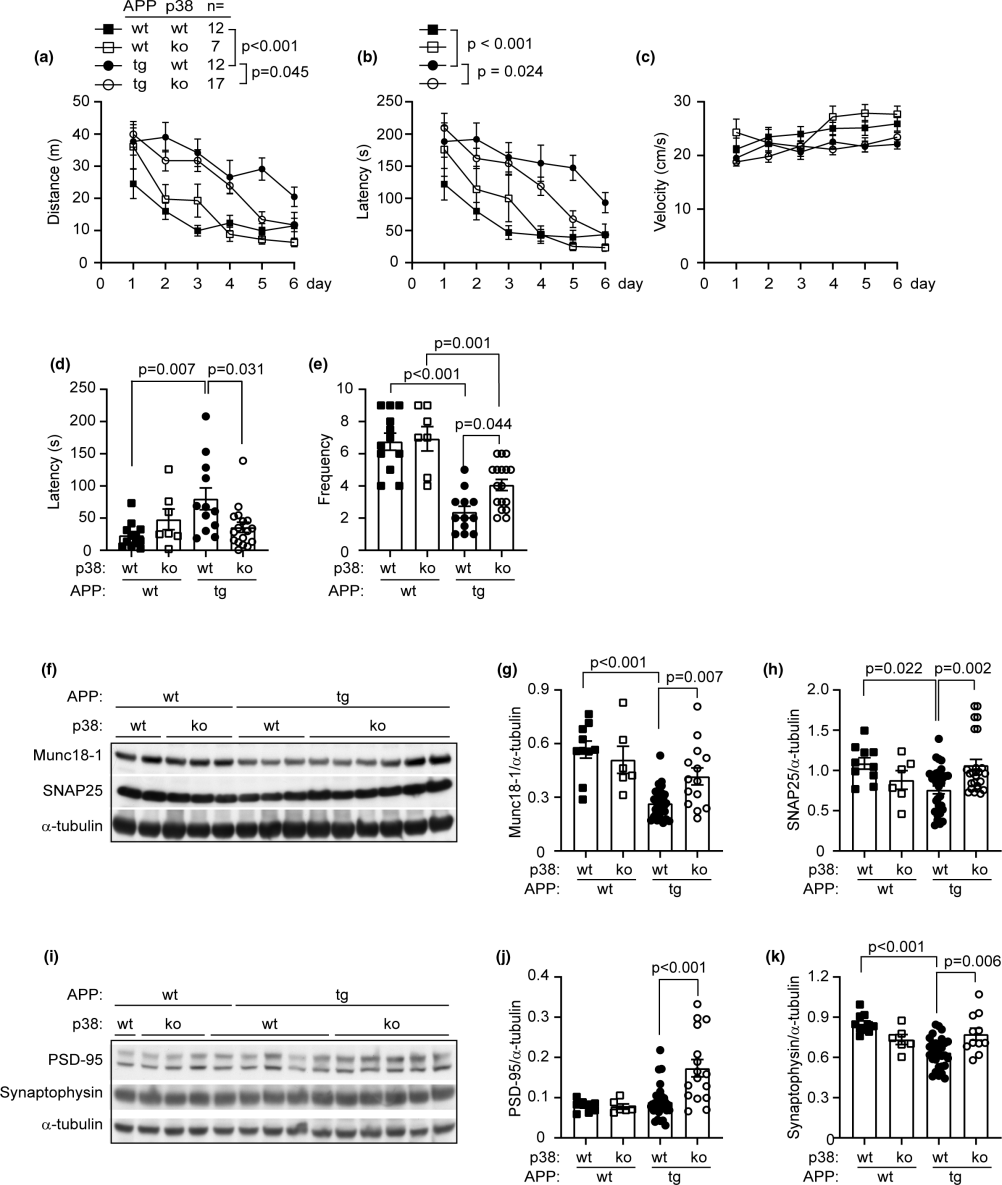
Deficiency of p38α‐MAPK in myeloid cells improves cognitive function and attenuates AD‐associated loss of synaptic proteins in APP‐transgenic mice. (a–c) Nine‐month‐old APP‐transgenic (APPtg) and non‐APP‐transgenic (APPwt) littermate mice with (p38α ko) and without (p38α wt) deletion of p38α‐MAPK in myeloid cells were assessed for cognitive function using the water maze test. In the training phase, deficiency of p38α‐MAPK decreases swimming distance (a) and latency (b) to reach the escape platform in APPtg but not in APPwt mice. Deficiency of p38α‐MAPK does not affect the traveling velocity in APPtg mice (c). Two‐way ANOVA from day 3 to day 6 followed by Bonferroni's post hoc test, *n* is shown in the figure. The latency of first visit to the region where the escape platform was previously located (d) and the frequency, with which mice crossed the platform region (e), were recorded in the 5‐min probe trial. One‐way ANOVA followed by Bonferroni's post hoc test. (f–k) The amount of synaptic proteins, Munc18‐1, SNAP25, synaptophysin, and PSD‐95 in the brain homogenate of 9‐month‐old APPtg and APPwt mice was determined using Western blotting. One‐way ANOVA followed by Bonferroni's post hoc test, *n* ≥ 11 per group for APPtg mice and *n* ≥ 6 per group for APPwt mice. Here, representative Western blot images from five independent experiments are shown. Munc18‐1 and SNAP15, PSD‐95 and synaptophysin, and their corresponding α‐tubulin immunoblots were performed on the same membrane. Data was represented as mean ± *SEM*

We used the Morris water maze test to examine cognitive function of 9‐month‐old APP^tg^ and their non‐APP‐transgenic (APP^wt^) littermate mice. During the acquisition phase, APP^wt^ mice with or without deletion of p38α‐MAPK in myeloid cells (APP^wt^p38α^fl/fl^LysM‐Cre^+/−^ and APP^wt^p38α^fl/fl^LysM‐Cre^−/−^) showed no significant differences in either swimming time or swimming distance before climbing onto the escape platform (Figure 1a,b). Compared to APP^wt^p38α^fl/fl^LysM‐Cre^−/−^ littermates, 9‐month‐old APP^tg^p38α^fl/fl^LysM‐Cre^−/−^ mice with normal p38α‐MAPK expression travelled significantly longer distances (Figure 1a) and spent significantly more time (Figure 1b) to reach the escape platform. Interestingly, APP^tg^p38α^fl/fl^LysM‐Cre^+/−^ mice with the deletion of myeloid p38α‐MAPK performed significantly better than their APP^tg^p38α^fl/fl^LysM‐Cre^−/−^ littermates in searching and finding the platform after 3 days of training (Figure 1a,b). The swimming velocity did not differ between p38α‐MAPK‐deficient and wildtype APP‐transgenic mice or for the same mice on different training dates (Figure 1c).

Twenty‐four hours after the end of training phase, the escape platform was removed and a 5‐min probe trial was performed to test the memory of mice. Compared to APP^wt^p38α^fl/fl^LysM‐Cre^−/−^ littermates, APP^tg^p38α^fl/fl^LysM‐Cre^−/−^ mice remained for a significantly longer time in their first visit to the region where the platform had been located, and crossed the original platform region with significantly less frequency during the total 5‐min probe trial (Figure 1d,e). Interestingly, when compared to APP^tg^p38α^fl/fl^LysM‐Cre^−/−^ mice, APP^tg^p38α^fl/fl^LysM‐Cre^+/−^ mice were able to reach the original platform region in significantly less time and crossed the region more frequently (Figure 1d,e). We observed differences in neither parameter analyzed in the probe trial between APP^wt^p38α^fl/fl^LysM‐Cre^+/−^ and APP^wt^p38α^fl/fl^LysM‐Cre^−/−^ littermate mice (Figure 1d,e).

We further used Western blot analysis to quantify the levels of four synaptic proteins: Munc18‐1, synaptophysin, SNAP‐25, and PSD‐95 in the brain homogenate of 9‐month‐old APP^tg^ and APP^wt^ littermate mice. As shown in Figure 1f–k, protein levels of Munc18‐1, synaptophysin and SNAP‐25 in APP^tg^p38α^fl/fl^LysM‐Cre^−/−^ mice were significantly lower than levels of these proteins derived from APP^wt^p38α^fl/fl^LysM‐Cre^−/−^ littermate mice. The reduction in Munc18‐1, synaptophysin and SNAP‐25 proteins due to APP‐transgenic expression was rescued by the deletion of p38α‐MAPK in myeloid cells (Figure 1g,h,k). PSD‐95 protein levels were significantly higher in brains from APP^tg^p38α^fl/fl^LysM‐Cre^+/−^ mice than that from APP^tg^p38α^fl/fl^LysM‐Cre^−/−^ control mice (Figure 1j). Comparison of APP^wt^p38α^fl/fl^LysM‐Cre^+/−^ and APP^wt^p38α^fl/fl^LysM‐Cre^−/−^ littermate mice showed no significant differences in protein levels of these four tested synaptic proteins (Figure 1f–k).

### 2.3 Deficiency of p38α‐MAPK in myeloid cells reduces Aβ load in the brain of APP‐transgenic mice

**FIGURE 2:**
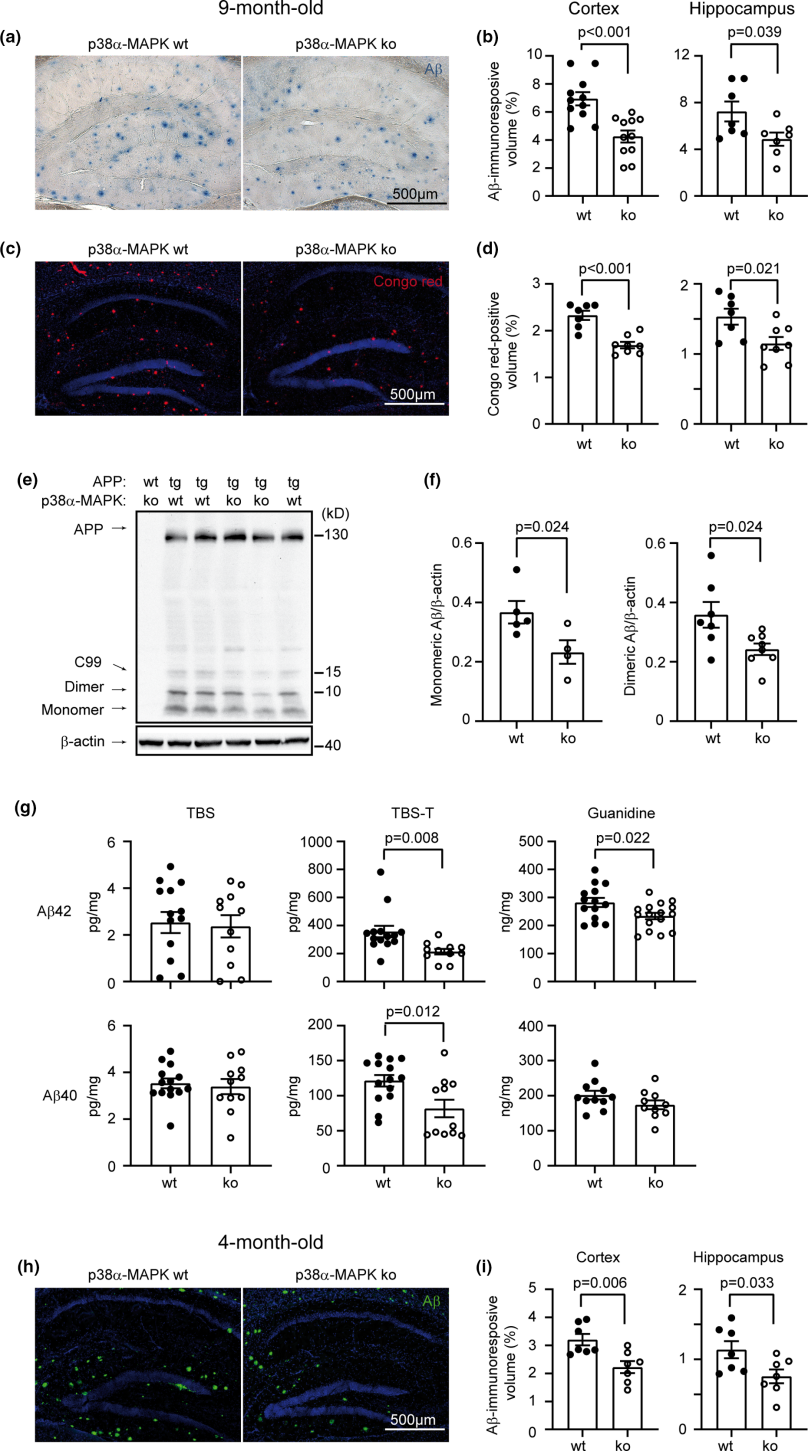
Deficiency of p38α‐MAPK in myeloid cells reduces Aβ load in the brain of APP‐transgenic mice. Four and 9‐month‐old APP‐transgenic mice with (p38α ko) and without (p38α wt) deletion of p38α‐MAPK in myeloid cells were analyzed with stereological *Cavalieri* methods for cerebral Aβ volumes (adjusted by the volume of analyzed tissues) after immunohistochemical (a, b), Congo red (c, d) and immunofluorescent (h, i) staining. *T* test, *n* ≥ 7 per each group. (e, f) Aβ was also detected in brain homogenates of 9‐month‐old p38α‐MAPK‐ko and ‐wt APP‐transgenic mice with Western blot. Here, representative Western blot images from two independent experiments are shown. Aβ and β‐Actin immunoblots were performed on the same membrane. *T* test, *n* ≥ 4 per each group. (g) APP‐transgenic mouse brains were further serially homogenized in TBS‐, TBS‐T‐, and guanidine‐soluble fractions, in which monomeric, oligomeric and high‐molecular‐weight Aβ aggregates were enriched, respectively. Aβ40 and Aβ42 were measured by ELISA and normalized to the amount of homogenate protein. *T* test, *n* ≥ 10 per group. Data was represented as mean ± *SEM*

As Aβ is the key molecule leading to neurodegeneration in AD (Scheltens et al., 2021), we analyzed the effects of myeloid p38α‐MAPK on Aβ pathology in the APP‐transgenic mice. Using immunohistological and stereological *Cavalieri* methods, we observed that the volume of immunoreactive Aβ load in 9‐month‐old APP^tg^p38α^fl/fl^LysM‐Cre^+/−^ mice was significantly lower than that in APP^tg^p38α^fl/fl^Cre^−/−^ littermate mice (Figure 2a,b). The brain tissue was also stained with Congo red that typically binds to the β sheet structure of Aβ plaques, which showed that deficiency of p38α‐MAPK decreased the cerebral level of Aβ aggregates (Figure 2c,d), corroborating the results from immunohistochemistry.

The amount of differently aggregated Aβ in brain tissue homogenates was measured with Western blot (The establishment of method was shown in Figure S3) and ELISA. Protein levels of monomeric and dimeric Aβ in 9‐month‐old APP^tg^p38^fl/fl^LysM‐Cre^+/−^ mice were significantly lower than that in APP^tg^p38^fl/fl^LysM‐Cre^−/−^ littermates (Figure 2e,f). Similarly, p38α‐MAPK deficiency significantly decreased concentrations of both Aβ40 and Aβ42 in TBS plus 1% Triton X‐100 (TBS‐T)‐soluble, and Aβ42 in guanidine hydrochloride‐soluble brain homogenates of APP^tg^p38^fl/fl^LysM‐Cre^+/−^ mice compared with APP^tg^p38^fl/fl^LysM‐Cre^−/−^ littermate mice (Figure 2g).

In order to learn the effects of myeloid p38α‐MAPK on Aβ pathology during the disease progression, Aβ deposits in 4‐month‐old APP^tg^p38α^fl/fl^LysM‐Cre^+/−^ and APP^tg^p38α^fl/fl^LysM‐Cre^−/−^ mice were also analyzed. Deletion of p38α‐MAPK in myeloid cells already reduced Aβ deposits in APP‐transgenic mouse brain at this early disease stage compared with p38α‐MAPK‐wildtype APP‐transgenic mice (Figure 2h,i); however, Western blot did not show significant effects of p38α‐MAPK deficiency on cerebral oligomeric Aβ levels (Figure S4).

### 2.4 Deficiency of myeloid p38α‐MAPK differently regulates microglial inflammatory activation in early and late disease stages of APP‐transgenic mice

**FIGURE 3:**
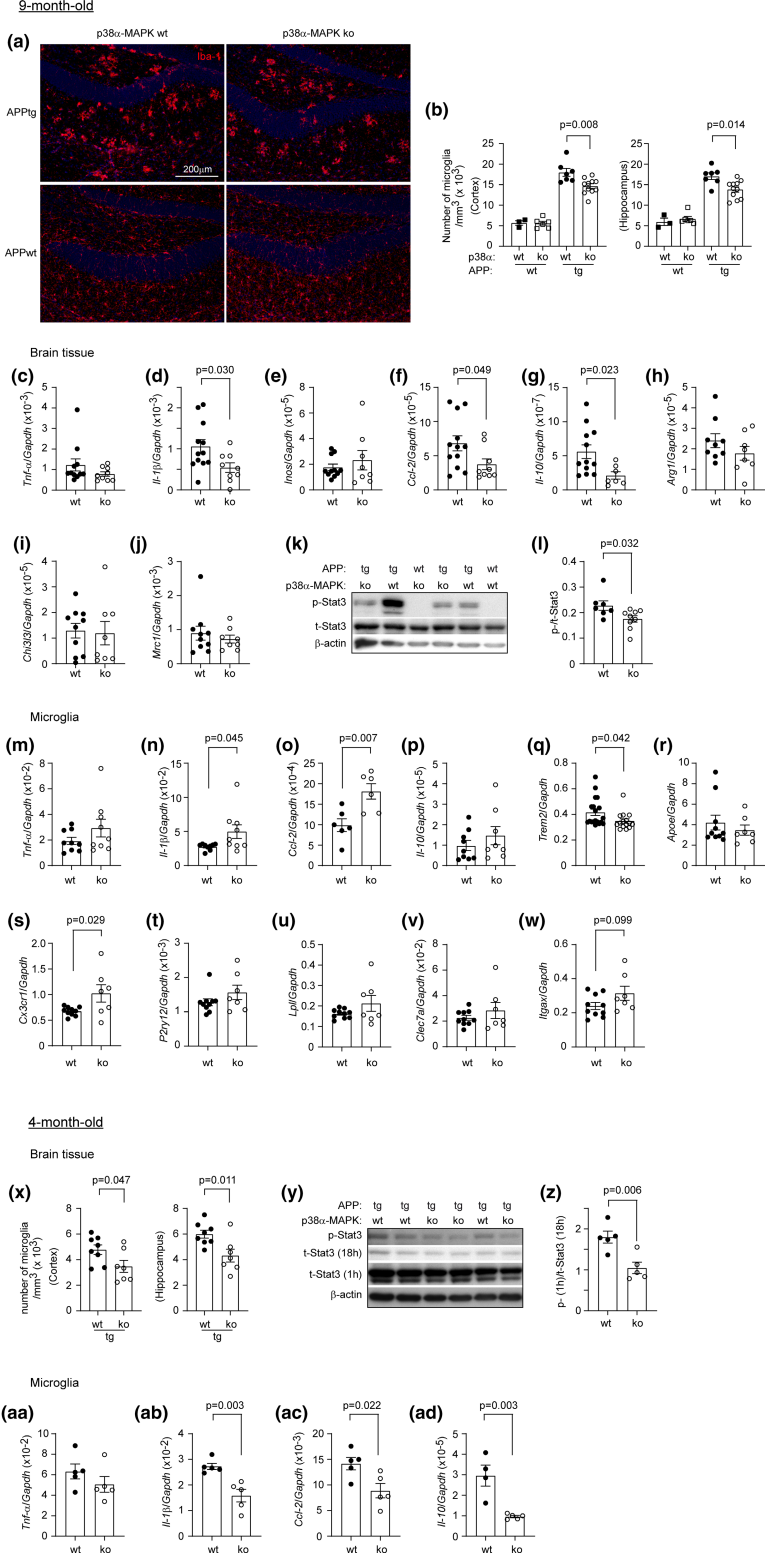
Deficiency of p38α‐MAPK in myeloid cells differently regulates microglial inflammatory activation in the brain of APP‐transgenic mice at early and late disease stages. (a, b, x) microglia stained with red fluorescent Iba‐1 antibody were counted with the optical fractionator stereological probe in brains of 4 and 9‐month‐old APP‐transgenic (APPtg) and non‐transgenic (APPwt) mice with (p38α‐ko) and without (p38α‐wt) deletion of p38α‐MAPK in myeloid cells. The cell number was adjusted by the volume of analyzed tissues. One‐way ANOVA followed by Bonferroni's post hoc test for 9‐month‐old mice, *n* ≥ 7 per group for APPtg mice and ≥3 per group for APPwt mice; *t* test for 4‐month‐old mice, *n* ≥ 7 per group. (a) Images show the immunofluorescent staining of 9‐month‐old mouse brains. (c–j) The inflammatory gene transcripts in brains of 9‐month‐old APPtg mice were measured with real‐time PCR. *T* test, *n* ≥ 8 per group. (k, l, y, z) four and nine‐month‐old APP^tg^p38^fl/fl^LysCre^+/−^ and APP^tg^p38^fl/fl^LysM‐Cre^−/−^ mice were further analyzed with Western blot for the levels of phosphorylated (Tyr705; p‐) and total (t‐) Stat3 in the brain. The same membrane was serially blotted with antibodies against p‐Stat3, t‐Stat3 and β‐Actin. The activity of Stat3 is shown in the ratio of p‐/t‐Stat3. *T* test, *n* ≥ 7 per group for 9‐month‐old mice and *n* = 5 per group for 4‐month‐old mice. (y) To avoid overexposure of the film, the membrane for t‐Stat3 blotting was additionally washed for 17 h after the first exposure to the film after 1 h of washing. Here, representative Western blot images from two independent experiments are shown. (m–w, aa–ad) in following experiments, microglia were selected from brains of 4‐ and 9‐month‐old p38α‐wt and ko APPtg mice. The transcriptional level of inflammatory genes and other DAM‐associated genes was determined by real‐time PCR. *T* test, *n* ≥ 6 and 4 per group for 9 and 4‐month‐old mice, respectively. Data was represented as mean ± *SEM*

Inflammatory activation of microglia is another pathogenic factor in AD (Scheltens et al., 2021). After immunofluorescent staining of Iba‐1, we used the stereological method, Optical Fractionator probe, to count microglia in the hippocampus and cortex. Deficiency of p38α‐MAPK in myeloid cells significantly decreased Iba‐1‐immunoreactive microglia in both 4 and 9‐month‐old APP‐transgenic, but not in 9‐month‐old non‐APP‐transgenic mice (Figure 3a,b,x). We also observed that deficiency of p38α‐MAPK decreased the number of P2RY12‐immunoreactive microglia in the hippocampus (Figure S5). It has been reported that P2RY12 is a more specific protein marker for endogenous microglia (McKinsey et al., 2020).

Deficiency of p38α‐MAPK in myeloid cells reduced *Il‐1β*, *Ccl‐2* and *Il‐10*, but not *Tnf‐α*, *Inos*, *Arg1*, *Chi3l3* and *Mrc1* gene transcripts in the brain of 9‐month‐old APP^tg^p38α^fl/fl^Cre^+/−^ mice compared with APP^tg^p38α^fl/fl^Cre^−/−^ littermates (Figure 3c–j). Notably, p38α‐MAPK decreased the transcriptional level of *Ccl‐2*, but not *Tnf‐α*, *Il‐1β*, and *Il‐10* in the brain of 9‐month‐old non‐APP‐transgenic mice (Figure S6a–d). In the brain of 4‐month‐old APP‐transgenic mice, p38α‐MAPK deficiency changed the transcription of neither pro‐ (*Tnf‐α*, *Il‐1β*, *Inos*, and *Ccl‐2*) nor anti‐inflammatory genes (*Il‐10*, *Chi3l3*, and *Mrc1*; Figure S7a–g).

IL‐10 activates Stat3. The levels of phosphorylated Stat3 in both 4‐ and 9‐month‐old APP^tg^p38^fl/fl^LysM‐Cre^+/−^ mice were significantly lower than that in APP^tg^p38^fl/fl^LysM‐Cre^−/−^ littermate controls (Figure 3k,l,y,z). Phosphorylated Stat3 was undetectable in the brain of 9‐month‐old APP^wt^ mice (Figure 3k), suggesting that IL‐10/Stat3‐mediated inflammatory signaling was activated in the brain of APP‐transgenic mice and inhibited by p38α‐MAPK deficiency in myeloid cells in both early and late disease stages.

To further analyze the inflammatory activity of microglia in p38α‐MAPK‐deficient AD mice, we isolated CD11b^+^ microglia from both 4‐ and 9‐month‐old APP‐transgenic mouse brains and detected inflammatory gene transcripts. Surprisingly, deficiency of p38α‐MAPK in myeloid cells significantly reduced the transcription of *Il‐1β*, *Ccl‐2* and *Il‐10* genes in cells from 4‐month‐old APP‐transgenic mice (Figure 3ab–ad), but increased the transcription of *Il‐1β* and *Ccl‐2* genes in cells from 9‐month‐old APP‐transgenic mice (Figure 3n,o), compared with p38α‐MAPK‐wildtype APP mice. Transcription of other tested inflammatory genes, for example, *Tnf‐α* in both 4 and 9‐month‐old APP‐transgenic mice (Figure 3m,aa) and *Il‐10* in 9‐month‐old APP‐transgenic mice (Figure 3p), was not altered by p38α‐MAPK deficiency. In microglia isolated from 9‐month‐old APP^wt^ mice, p38α‐MAPK deficiency did not alter transcripts of all tested genes, *Tnf‐α*, *Il‐1β*, *Ccl‐2* and *Il‐10* genes (Figure S6e–h).

Regarding other molecular signatures of disease‐associated microglia (DAM; Keren‐Shaul et al., 2017), we observed a significant increase of *Cx3cr1* transcription (Figure 3s), and a decrease of *Trem2* transcription (Figure 3q) in CD11b + microglia from 9‐month‐old APP^tg^p38α^fl/fl^LysM‐Cre^+/−^ mice compared with APP^tg^p38α^fl/fl^LysM‐Cre^−/−^ littermates. Deficiency of p38α‐MAPK did not affect the transcription of *Apoe*, *P2ry12*, *Lpl*, *Clec7a* and *Itgax* genes in microglia of 9‐month‐old APP‐transgenic mice (Figure 3r,t–w). In 4‐month‐old APP‐transgenic mice, transcription of none of the DAM‐associated genes tested in 9‐month‐old mice was altered by the deficiency of p38α‐MAPK in myeloid cells (Figure S7h–n).

### 2.5 Deficiency of myeloid p38α‐MAPK increases microglial clearance of Aβ in APP‐transgenic mice at the late disease stage

**FIGURE 4:**
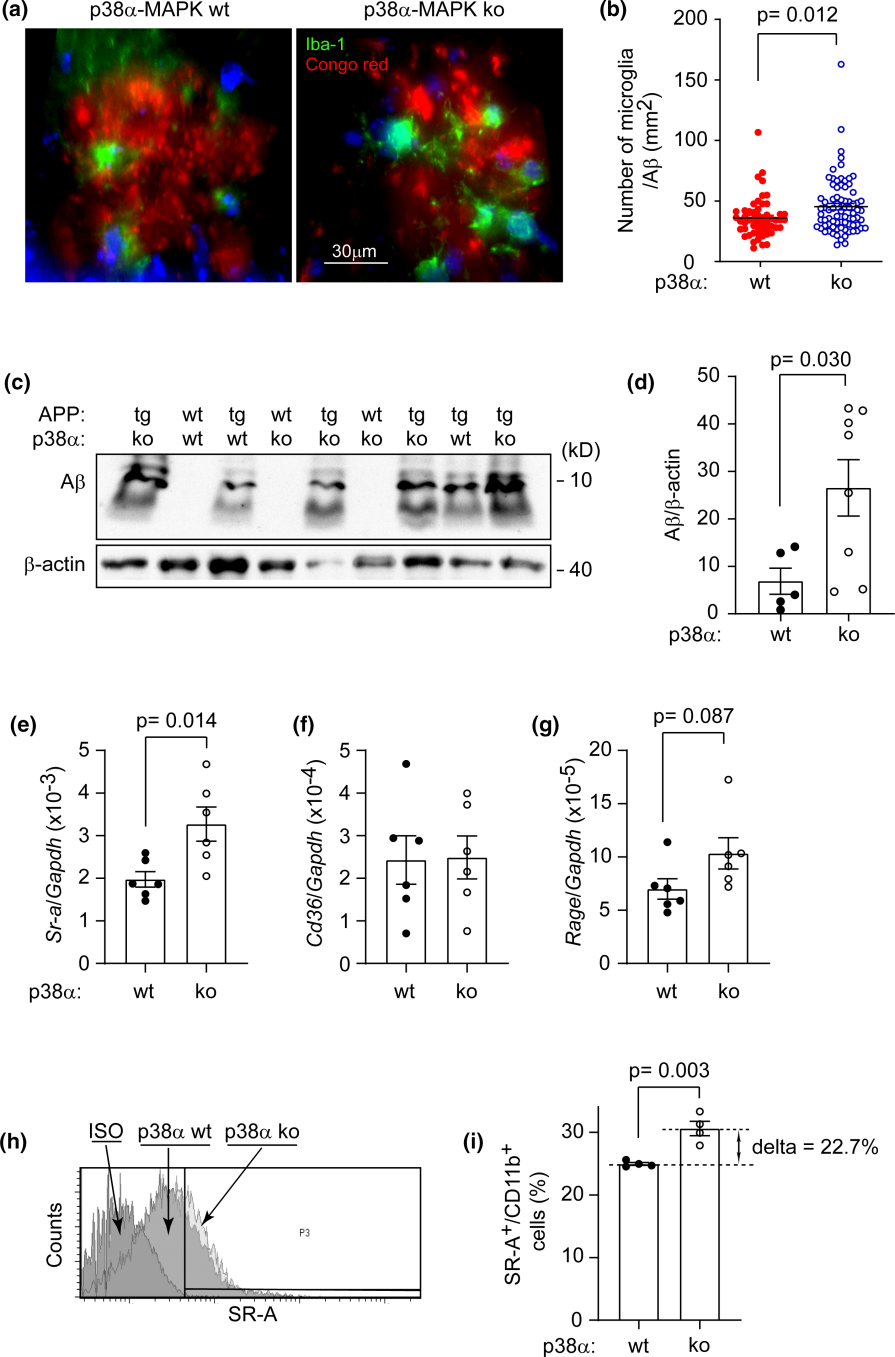
Deficiency of p38α‐MAPK in myeloid cells promotes microglial internalization of Aβ in the brain of 9‐month‐old APP‐transgenic mice. (a, b) Brain sections from 9‐month‐old p38α‐MAPK‐deficient (ko) and wildtype (wt) mice were stained for microglia with green fluorescent Iba‐1 antibodies and for Aβ deposits with Congo red. Under the red channel, total 105 Aβ deposits in p38α‐MAPK‐ko mice and 76 Aβ deposits in p38α‐MAPK‐wt mice were randomly chosen. Microglia with clear DAPI‐stained nuclei and with contact to Aβ deposits were counted. The number of microglia was adjusted by the area of Congo red‐positive Aβ deposits. *T* test, *n* = 5 and 4 for p38α‐MAPK‐ko and wt APP‐transgenic mice, respectively. (c, d) Adult microglia were also isolated from 9‐month‐old p38α‐MAPK‐ko and wt APP‐transgenic mice, and quantified for intracellular Aβ by Western blot using human Aβ and β‐Actin antibodies. As a control, no Aβ was detected in the microglia isolated from APP‐wildtype mice (c). Here, representative Western blot images from three independent experiments are shown. Aβ and β‐Actin immunoblots were performed on the same membrane. The overall picture of the Aβ‐immunoblot is shown in Figure S3c. *T* test, *n* ≥ 5 per group. (e–g) The microglial gene transcription of Aβ internalization‐associated receptors, such as SR‐A, CD36, and RAGE, in 9‐month‐old APP‐transgenic mice was detected with real‐time PCR. *T* test, *n* ≥ 6 per group. (h, i) The protein level of SR‐A on microglia was determined by flow cytometry after immunofluorescent staining of SR‐A. *t* test, *n* = 4 per group. Data was represented as mean ± *SEM*

Microglia play like a double‐edged sword. Their uptake of Aβ is an important mechanism of Aβ clearance in AD brain (Scheltens et al., 2021). We asked whether deficiency of p38α‐MAPK facilitates microglial internalization of Aβ in AD mice. After observing that there were more microglia surrounding Aβ deposits in 9‐month‐old APP^tg^p38^fl/fl^LysM‐Cre^+/−^ mice than in APP^tg^p38^fl/fl^LysM‐Cre^−/−^ littermate mice (Figure 4a,b), we isolated microglia from these two groups of AD mice and quantified intracellular Aβ with Western blot (Figure S3b,c). As shown in Figure 4c,d, the protein level of intracellular Aβ in p38α‐MAPK‐deficient cells was higher than that in p38α‐MAPK‐wildtype microglia. Furthermore, we evaluated expression levels of Aβ internalization‐associated receptors in microglia with quantitative RT‐PCR and flow cytometry. Deficiency of p38α‐MAPK increased expression of scavenger receptor A (SR‐A) in microglia at both transcriptional and protein levels compared with microglia from p38α‐MAPK‐wildtype APP‐transgenic mice (Figure 4e,h,i). The transcription of other Aβ internalization‐associated receptors, such as CD36 and RAGE, was not changed by deficiency of p38α‐MAPK (Figure 4f,g).

In 4‐month‐old APP‐transgenic mice, we repeated all experiments for 9‐month‐old mice. We observed that p38α‐MAPK deficiency neither altered the intracellular Aβ in microglia, nor affected the transcription of Aβ internalization‐associated receptors, including SR‐A, CD36, and RAGE (Figure S1d and Figure S8).

In order to verify our in vivo observation that p38α‐MAPK deficiency enhances Aβ internalization in microglia, we cultured p38α‐MAPK‐deficient and wildtype bone marrow‐derived macrophages (BMDMs) and primed them with and without 100 ng/ml lipopolysaccharide (LPS) for 48 h. Deficiency of p38α‐MAPK did increase Aβ internalization in inflammatorily activated macrophages in association with an up‐regulation of SR‐A, but not in resting cells (Figure S9a–f). Co‐treatment with fucoidan, an antagonist of SR‐A, abolished p38α‐MAPK deficiency‐enhanced Aβ internalization (Figure S9g,h).

### 2.6 Deficiency of p38α‐MAPK specifically in microglia reduces AD‐associated pathologies in the brain of APP‐transgenic mice, but with low efficiency

**FIGURE 5:**
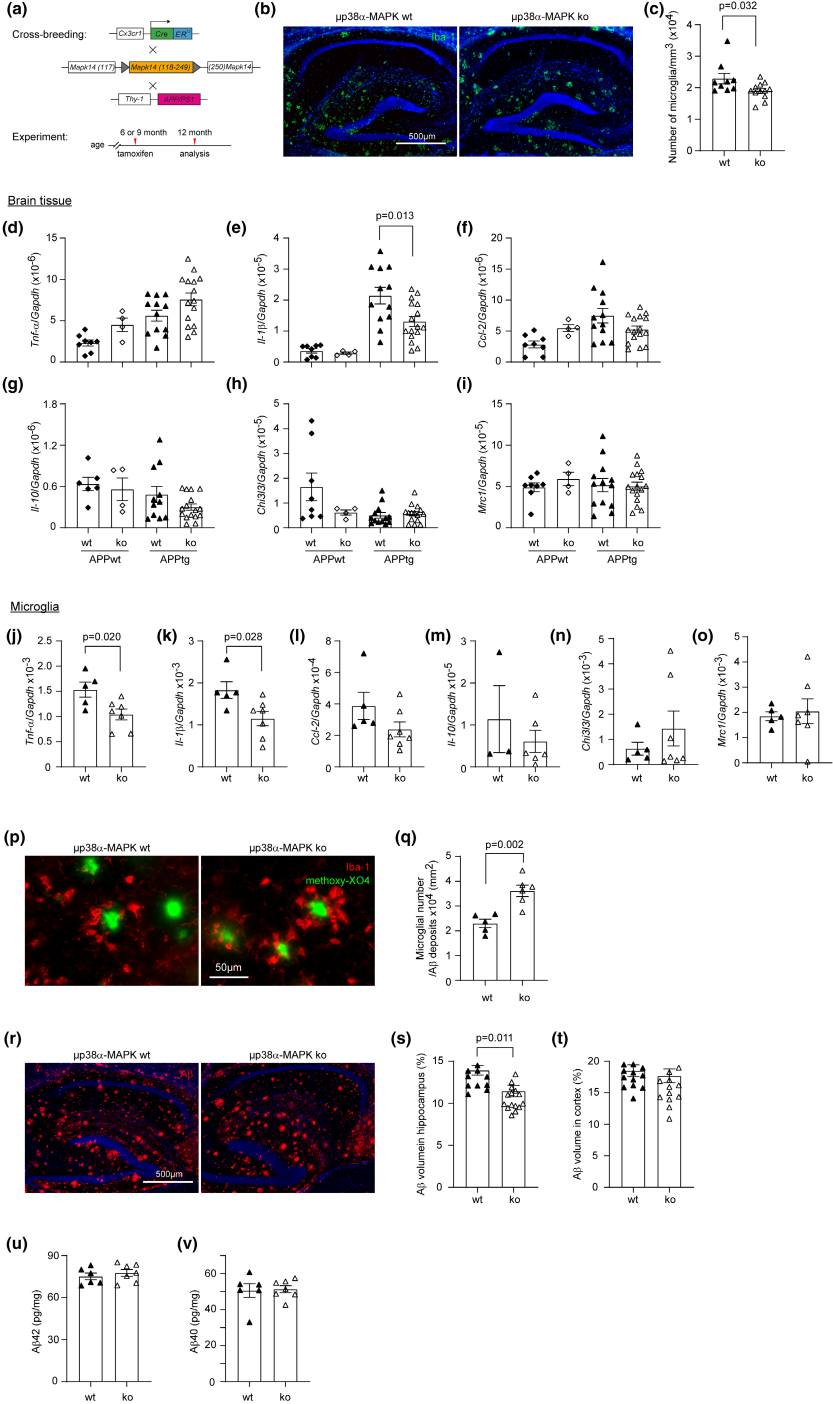
Deficiency of p38α‐MAPK specifically in microglia inhibits inflammatory activation and decreases Aβ load in the brain of APP‐transgenic mice. (a) APP^tg^p38^fl/fl^Cx3Cr1‐Cre^+/−^ and APP^tg^p38^fl/fl^Cx3Cr1‐Cre^−/−^ littermate mice were injected with tamoxifen at 6 or 9 months of age and analyzed at 12 months. (b, c) Microglia in the hippocampus of microglial p38α‐MAPK‐deficient (μp38α‐MAPK‐ko) and wildtype (μp38α‐MAPK‐wt) APP‐transgenic (APPtg) mice were stained with green fluorescence‐conjugated Iba‐1 antibodies and counted with the optical fractionator probe. The number of microglia was adjusted by the volume of analyzed brain tissue. *T* test, *n* ≥ 9 per group. (d–i) Inflammatory gene transcripts in brain tissues were measured with real‐time PCR. As a control, non‐APP‐transgenic (APPwt) littermates were treated with tamoxifen, as in APPtg mice, to induce deletion of p38α‐MAPK in microglia. One‐way ANOVA followed by Bonferroni's post hoc test, *n* ≥ 11 and 4 per group for APPtg and APPwt mice, respectively. (j–o) CD11b+ cells were further selected from brains of 12‐month‐old APPtg mice. Transcripts of various inflammatory genes in microglia were quantified with real‐time RT‐PCR. *T* test, *n* ≥ 5 per group. (p, q) in following experiments, brain sections of 12‐month‐old μp38α‐wt and ‐ko APPtg mice were stained with red fluorescent Iba‐1 antibodies for microglia and with methoxy‐XO4 (in green) for Aβ deposits. Microglia around Aβ deposits were counted and the number of microglia was adjusted by the area of Aβ deposits. *T* test, *n* ≥ 5 per group. (r–t) Finally, the coverage of Aβ deposits in the brain as stained by human Aβ antibodies was estimated with *Cavalieri* method and adjusted by the area of analyzed brain tissue. *T* test, *n* ≥ 10 per group. (u, v) Aβ40 and Aβ42 in RIPA‐soluble brain homogenates of 12‐month‐old APPtg mice with and without deletion of p38α‐MAPK in microglia were measured with ELISA. *T* test, *n* ≥ 6. Data was represented as mean ± *SEM*

After observing that deficiency of p38α‐MAPK in whole myeloid cells prevented AD progression, we asked whether p38α‐MAPK deficiency specifically in microglia served the same beneficial effects. A second AD mouse model was constructed by cross‐breeding APP^tg^ mice with p38^fl/fl^ mice and Cx3Cr1‐CreERT2 mice as we did in a recent study (Quan et al., 2021). Six or nine‐month‐old APP^tg^p38^fl/fl^Cx3Cr1‐Cre^+/−^ and APP^tg^p38^fl/fl^Cx3Cr1‐Cre^−/−^ littermate mice were injected with tamoxifen, and analyzed at 12 months of age (Figure 5a). Tamoxifen injection induced p38α‐MAPK deficiency in both microglia and Cx3Cr1‐positive peripheral myeloid cells; however, normal p38α‐MAPK‐expressing myeloid cells produced from the bone marrow replaced the peripheral p38α‐MAPK‐deficient myeloid cells within 1 month (Goldmann et al., 2013). The efficiency of tamoxifen‐induced gene recombination was 97% in CD11b + brain cells of APP^tg^p38^fl/fl^Cx3Cr1‐Cre^+/−^ mice (*Mapk14*/*Gapdh*: 0.007 ± 0.001 and 0.212 ± 0.035, in APP^tg^p38^fl/fl^Cx3Cr1‐Cre^+/−^ and APP^tg^p38^fl/fl^Cx3Cr1‐Cre^−/−^ mice, respectively; *t* test, *p* < 0.001), which was in accordance with a previous observation (Goldmann et al., 2013). As a control, the transcriptional level of *Mapk14* gene in CD11b + blood cells was not different between these two groups of mice (*Mapk14/Gapdh*: 3.522 ± 0.736 and 3.874 ± 0.800, in APP^tg^p38^fl/fl^Cx3Cr1‐Cre^+/−^ and APP^tg^p38^fl/fl^Cx3Cr1‐Cre^−/−^ mice, respectively; *t* test, *p = 0.761*).

Deletion of p38α‐MAPK in microglia of APP‐transgenic mice (APP^tg^p38^fl/fl^Cx3Cr1‐Cre^+/−^) at 6 months significantly reduced microglia in the hippocampus at 12 months compared with APP^tg^p38^fl/fl^Cx3Cr1‐Cre^−/−^ littermates (Figure 5b,c). Similarly, microglial deficiency of p38α‐MAPK significantly decreased the transcription of *Il‐1β* in the brain tissue from APP‐transgenic, but not APP‐wildtype mice (Figure 5e). Microglial deficiency of p38α‐MAPK did not change transcription of other inflammatory genes tested (e.g., *Tnf‐α*, *Ccl‐2*, *Il‐10*, *Chi3l3* and *Mrc1*; Figure 5d,f–i). To analyze the effect of p38α‐MAPK on inflammatory activation in individual microglia, we isolated microglia from 12‐month‐old APP^tg^p38^fl/fl^Cx3Cr1‐Cre^+/−^ and APP^tg^p38^fl/fl^Cx3Cr1‐Cre^−/−^ littermate mice injected with tamoxifen at 9 months of age. Deletion of p38α‐MAPK significantly decreased transcripts of *Tnf‐α* and *Il‐1β*, but not *Ccl‐2*, *Il‐10*, *Chi3l3* and *Mrc1* genes in microglia of APP‐transgenic mice (Figure 5j–o). It was different from in 9‐month‐old myeloid p38α‐MAPK‐deficient APP‐transgenic mice (see Figure 3m–w).

However, as the same as in 9‐month‐old APP^tg^p38^fl/fl^LysM‐Cre^+/−^ mice, p38α‐MAPK deficiency specifically in microglia also promoted the accumulation of Iba‐1‐positive microglia around Aβ deposits in both cortex and hippocampus of 12‐month‐old APP^tg^p38^fl/fl^Cx3Cr1‐Cre^+/−^ mice compared with p38α‐MAPK‐wildtype AD mice (Figure 5p,q). We also observed that deletion of p38α‐MAPK in microglia at 6 to 12 months of age significantly decreased Aβ deposition in the hippocampus but not in the cortex (Figure 5r–t). Concentrations of both Aβ40 and Aβ42 in RIPA‐soluble brain homogenates quantitated by ELISA did not differ between APP^tg^p38^fl/fl^Cx3Cr1‐Cre^+/−^ and APP^tg^p38^fl/fl^Cx3Cr1‐Cre^−/−^ mice (Figure 5u,v).

As APP^tg^p38^fl/fl^Cx3Cr1‐Cre^+/−^ mice were haploinsufficient for *Cx3cr1* gene, additional experiments were performed to examine whether Cx3Cr1 haploinsufficiency affects AD pathogenesis. Our recent study showed that Cx3Cr1 haploinsufficiency does not change Aβ deposition and inflammation in the brain of APP‐transgenic mice (Quan et al., 2021). Our current study indicated that haploinsufficiency of Cx3Cr1 altered neither the recruitment of microglia toward Aβ deposits, nor the transcription of inflammatory genes, *Tnf‐α*, *Il‐1β*, *Ccl‐2* and *Il‐10* in individual microglia (Figure S10).

In further experiments, we observed that microglial deficiency of p38α‐MAPK attenuated the cognitive deficits of 12‐month‐old APP‐transgenic mice in Morris water maze test; however, p38α‐MAPK deficiency did not prevent the loss of synaptophysin, Munc18‐1, PSD‐95, and SNAP25 in APP‐transgenic AD mice (Figure S11).

### 2.7 IL17a‐expressing lymphocytes might be involved in attenuating Aβ pathology in myeloid p38α‐MAPK‐deficient APP‐transgenic mice

The comparison of APP^tg^p38^fl/fl^LysM‐Cre^+/−^ and APP^tg^p38^fl/fl^Cx3Cr1‐Cre^+/−^ mouse models strongly suggested that peripheral p38α‐MAPK‐deficient myeloid cells were more efficient than p38α‐MAPK‐deficient microglia in reducing cerebral Aβ in AD mice. We hypothesized that deficiency of myeloid p38α‐MAPK cells regulates peripheral immune cells and indirectly affects brain pathology. Interestingly, 6‐month‐old APP‐transgenic mice showed that transcription of *Il‐17a*, but not *Ifn‐γ*, *Il‐4* and *Il‐10* genes, was significantly up‐regulated in CD4+ spleen cells compared with APP‐wildtype littermate mice (Figure S12a–d). By cross‐breeding APP^tg^ mice with IL‐17a‐eGFP reporter mice, in which eGFP is expressed under the control of endogenous *Il‐17a* gene promoter (Esplugues et al., 2011), and detecting GFP‐expressing lymphocytes in the intestine, we observed that there were significantly more GFP‐expressing CD4+ lymphocytes in both lamina propria and Peyer's patches of 6‐month‐old APP‐transgenic mice than in APP‐wildtype littermates (Figure S12e–i).

**FIGURE 6:**
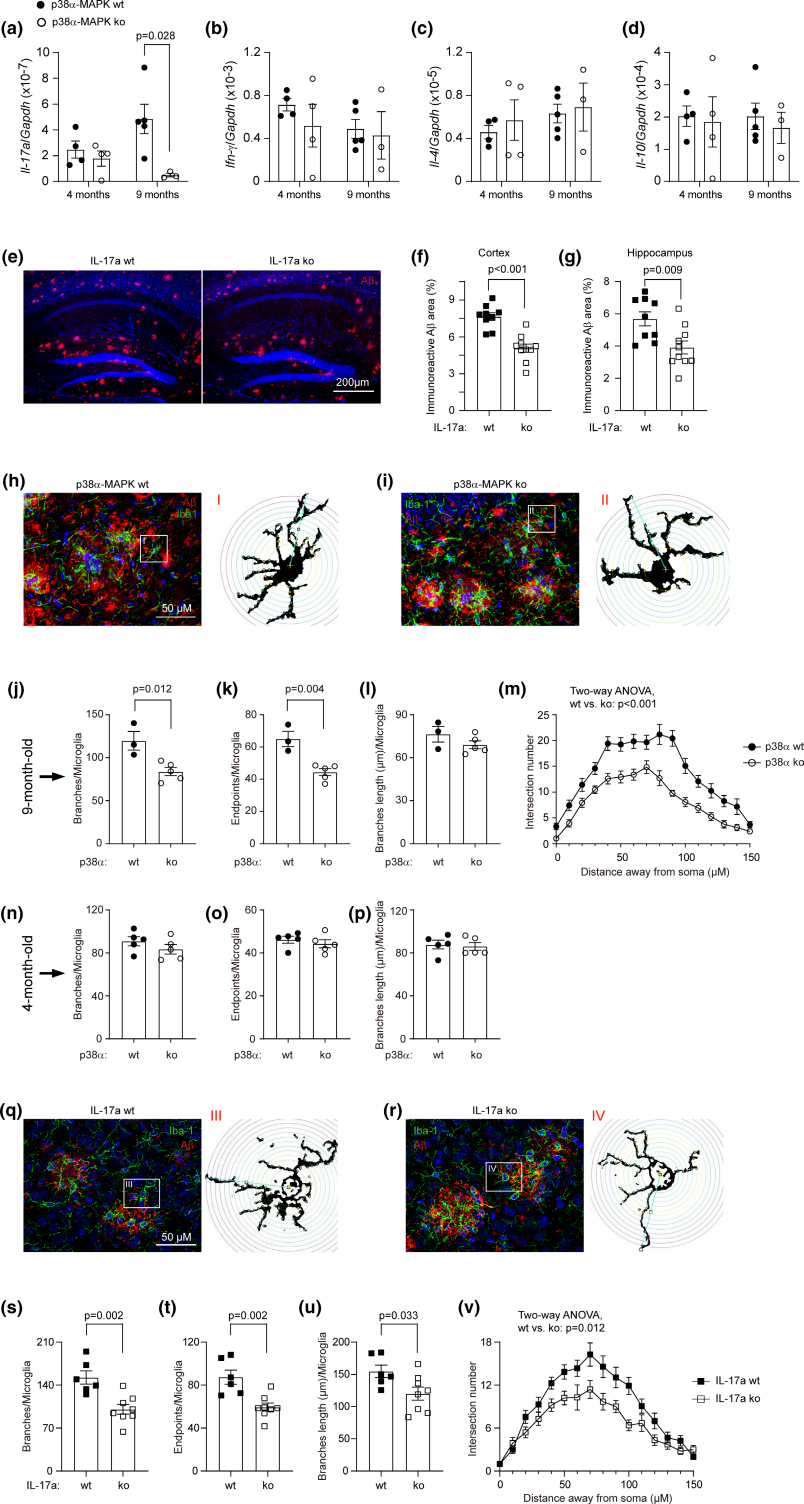
Deficiency of p38α‐MAPK in myeloid cells and knockout of IL‐17a similarly modify microglial morphology and reduce Aβ deposits in APP‐transgenic mice. (a–d) CD4‐positive spleen cells were selected from 4‐ and 9‐month‐old APP‐transgenic mice with (ko) and without (wt) deletion of p38α‐MAPK in myeloid cells. Real‐time PCR was used to quantify transcripts of marker genes for Th17 (*IL‐17a*), Th1 (*Ifn‐γ*), Th2 (*IL‐4*) and regulatory T (Treg) lymphocytes (*Il‐10*). *T* test, *n* ≥ 3 per group. (e–g) To investigate the pathogenic role of IL‐17a in AD, APP‐transgenic mice were mated to IL‐17a knockout mice. Brains of 6‐month‐old APP‐transgenic mice with (ko) and without (wt) knockout of IL‐17a were stained with antibodies against human Aβ (e). The volume of immunoreactive Aβ‐positive staining was estimated with stereological *Cavalieri* method and adjusted by the volume of analyzed brain tissue. *T* test, *n* ≥ 9 per group. (h–p) In following experiments, the morphology of microglia in contact with Aβ deposits was analyzed in 4 and 9‐month‐old p38α‐MAPK‐ko and ‐wt APP‐transgenic mice after immunofluorescent staining of Iba‐1 and Aβ (h, i, images from 9‐month‐old APP‐transgenic mice). The number of branches (j, n), endpoints of branches (k, o) and length of branches (l, p) of microglia were calculated and adjusted by the number of microglia. Deficiency of p38α‐MAPK in myeloid cells significantly decreases the number of branches, but not the length of branches of microglia in 9‐month‐old APP‐transgenic mice (j, k). *T* test, *n* ≥ 3 per group. (m) The Sholl analysis further shows that p38α‐MAPK deficiency decreases the number of microglial processes in 9‐month‐old APP‐transgenic mice. Total 14 microglia from five p38α‐MAPK‐ko mice and 12 microglia from three p38α‐MAPK‐wt mice were analyzed. Two‐way ANOVA testing the difference between p38α‐MAPK‐ko and ‐wt mice. (n–p) Deficiency of p38α‐MAPK in myeloid cells does not change the morphology of microglia in 4‐month‐old APP‐transgenic mice. *T* test, *p* > 0.05, *n* ≥ 3 per group. (q, r) Similarly, the morphology of microglia in contact with Aβ deposits was analyzed in 6‐month‐old IL‐17a‐ko and ‐wt APP‐transgenic mice after immunofluorescent staining of Iba‐1 and Aβ. (s–u) IL‐17a deficiency significantly reduces the number and total length of branches of microglia in APP‐transgenic mice compared with IL‐17a‐wt controls. *T* test, *n* ≥ 6 per group. (v) for the Sholl analysis, 10 microglia from 2 IL‐17a ko mice and 14 microglia from 2 IL‐17a wt mice were analyzed. Two‐way ANOVA was performed to test the difference between IL‐17a ko and wt mice. Data was represented as mean ± *SEM*

We then isolated CD4+ spleen cells from 4‐ and 9‐month‐old APP^tg^p38^fl/fl^LysM‐Cre^+/−^ and APP^tg^p38^fl/fl^LysM‐Cre^−/−^ littermate mice, and observed that p38α‐MAPK deficiency significantly reduced the transcription of *Il‐17a*, but not *Ifn‐γ*, *Il‐4* and *Il‐10* in AD mice at the age of 9, but not 4 months (Figure 6a–d). Thus, we hypothesized that IL‐17a‐expressing cells might be involved in cerebral Aβ reduction induced by peripheral p38α‐MAPK‐deficient myeloid cells.

We cross‐bred APP^tg^ mice with IL‐17a^−/−^ mice (Nakae et al., 2002) and observed that the extent of immunoreactive Aβ deposits in both the cortex and hippocampus of 6‐month‐old APP^tg^IL17a^−/−^ (IL‐17a knockout) mice was significantly less than that in APP^tg^IL17a^+/+^ (IL‐17a wildtype) littermates (Figure 6e–g).

To investigate whether IL‐17a deficiency models p38α‐MAPK‐deficient myeloid cells in regulating microglial activation, we analyzed and compared the morphology of microglia surrounding Aβ deposits. Deletion of p38α‐MAPK in myeloid cells decreased the total number and end points of branches of microglial processes in 9‐ but not 4‐month‐old APP‐transgenic mice (Figure 6h–p). In Sholl analysis, microglial branches crossed concentric circles significantly less in 9‐month‐old APP^tg^p38^fl/fl^LysM‐Cre^+/−^ than in APP^tg^p38^fl/fl^LysM‐Cre^−/−^ littermates, especially at 40–70 μm from the soma (Figure 6m). Interestingly, all changes of microglial morphology in 9‐month‐old APP^tg^p38^fl/fl^LysM‐Cre^+/−^ mice relative to APP^tg^p38^fl/fl^LysM‐Cre^−/−^ littermates could be produced in 6‐month‐old APP^tg^IL17a^−/−^ mice compared with APP^tg^IL17a^+/+^ mice (Figure 6q–v).

## 3 DISCUSSION

We constructed two AD mouse models with deletion of p38α‐MAPK in all myeloid cells (APP^tg^p38^fl/fl^LysM‐Cre^+/−^) and specifically in microglia (APP^tg^p38^fl/fl^Cx3Cr1‐Cre^+/−^). In APP^tg^p38^fl/fl^LysM‐Cre^+/−^ mice, LysM‐Cre reduced *Mapk14* transcription in microglia by 45% by 9 months of age and in CD11b+ blood cells by 88% as early as 4 months of age. Twelve‐month‐old APP^tg^p38^fl/fl^Cx3Cr1‐Cre^+/−^ mice showed a 97% decrease in *Mapk14* transcription in microglia, whereas there was no change in peripheral myeloid cells. Of note, deletion of p38α‐MAPK attenuated cerebral Aβ and neuronal damage in APP^tg^p38^fl/fl^LysM‐Cre^+/−^ mice but had little effect on these two pathologies in APP^tg^p38^fl/fl^Cx3Cr1‐Cre^+/−^ mice. Thus, it may be peripheral p38α‐MAPK‐deficient myeloid cells rather than p38α‐MAPK‐deficient microglia that can effectively prevent disease progression in APP‐transgenic mice.

Deficiency of p38α‐MAPK in myeloid cells inhibited inflammatory activation in individual microglia early in disease (by 4 months), but enhanced it after disease progression (by 9 months). The decrease of inflammatory gene transcripts (e.g., *Il‐1β* and *Ccl‐2*) in the whole brain of 9‐month‐old APP^tg^p38^fl/fl^LysM‐Cre^+/−^ mice was likely due to the decreased number of microglia beginning earlier in the disease (e.g., at 4 months of age). Interestingly, microglia internalized more Aβ in APP^tg^p38^fl/fl^LysM‐Cre^+/−^ mice than in APP^tg^p38^fl/fl^LysM‐Cre^−/−^ littermates at 9 but not 4 months of age. In cell cultures, we observed that p38α‐MAPK deficiency increased Aβ uptake by LPS‐primed cultured macrophages. Thus, p38α‐MAPK deficiency could promote microglial Aβ clearance in the context of inflammatory activation. Part of the possible mechanisms is that deficiency of p38α‐MAPK upregulates the expression of SR‐A, a typical Aβ‐phagocytic receptor (Paresce et al., 1996), in microglia. We also observed that p38α‐MAPK deficiency in myeloid cells inhibited IL‐10‐Stat3 signaling in the brain of APP‐transgenic mice. It has been reported that deficiency of IL‐10 or Stat3 facilitates microglial clearance of Aβ in AD mice (Guillot‐Sestier et al., 2015; Reichenbach et al., 2019). In APP^tg^p38^fl/fl^Cx3Cr1‐Cre^+/−^ mice, p38α‐MAPK deficiency inhibited inflammatory activation in microglia, which may prevent p38α‐MAPK deficiency from enhancing Aβ internalization. Indeed, mild inflammatory activation has the potential to increase Aβ clearance in the brain. Systemic injection of TLR4 or TLR9 agonists induces both pro‐ and anti‐inflammatory activation and decreases Aβ in the brain of APP‐transgenic mice (Michaud, Halle, et al., 2013; Scholtzova et al., 2014). TREM2 antibody administration also decreases Aβ load in the presence of increased expression of inflammatory cytokines and chemokines in the brain of APP‐transgenic mice (Price et al., 2020). However, the mechanisms of inflammatory regulation of microglial Aβ clearance remain unclear. The gene transcription in microglia from our APP^tg^p38^fl/fl^LysM‐Cre^+/−^ mice showed partial DAM signatures (e.g., induction of proinflammatory genes); however, transcription of homeostatic genes (e.g., *Cx3cr1*) was also up‐regulated and transcription of *Trem2* gene was reduced.

Our study was not yet able to answer the question of how peripheral p38α‐MAPK‐deficient myeloid cells reduced cerebral Aβ in 4‐month‐old APP^tg^p38^fl/fl^LysM‐Cre^+/−^ mice. The decrease in microglia at 4 months of age may be secondary to the Aβ reduction. It is generally accepted that microglia play a primary role in AD pathogenesis. However, AD is a systemic disease associated with dysregulation of the peripheral immune system. Peripheral myeloid cells have been reported to directly clear Aβ in blood vessels (Michaud, Bellavance, et al., 2013). Our cell culture experiments showed that p38α‐MAPK‐deficient macrophages took up more Aβ42 oligomers than p38α‐MAPK‐wildtype cells. In the following study, we generate bone marrow‐chimeric AD mice as we have done previously (Hao et al., 2011) by transplanting p38α‐MAPK‐deficient and wildtype hemopoietic stem cells into APP‐transgenic mice that have received selective‐body irradiation (omitting the brain). This experiment may answer the question whether deficiency of p38α‐MAPK in peripheral myeloid cells is sufficient to reduce the cerebral Aβ load.

It remains unclear how peripheral p38α‐MAPK‐deficient myeloid cells regulate the inflammatory activation of microglia. Since the number of cells expressing CD11b and CCR2‐RFP reporter in the brain did not differ between APP^tg^p38^fl/fl^LysM‐Cre^+/−^ and APP^tg^p38^fl/fl^LysM‐Cre^−/−^ littermate mice, it is unlikely that peripheral p38α‐MAPK‐deficient myeloid cells migrate into the brain parenchyma and interact directly with microglia. We observed that p38α‐MAPK‐deficient myeloid cells decreased *Il‐17a* gene transcription in CD4‐positive splenocytes at nine but not 4 months of age, which was correlated with changes in microglial inflammatory activation and morphology and Aβ internalization. The morphology of microglia surrounding Aβ deposits in nine (but not 4)‐month‐old myeloid p38α‐MAPK‐deficient APP‐transgenic mice was characterized by a reduction in the overall length, branches and end points of the processes, which are markers of microglial maturation and activation (Erny et al., 2021), and may also indicate active Aβ internalization (Huang et al., 2021). Very interestingly, the morphological pattern of microglia in p38α‐MAPK‐deficient mice can be generated in IL‐17a‐deficient APP‐transgenic mice. It is known that p38α‐MAPK signaling in dendritic cells drives differentiation of T helper 17 (Th17) cells and sustains autoimmune inflammation (Huang et al., 2012). We have observed that APP is expressed in myenteric neurons of the gut (Semar et al., 2013) and is able to increase IL‐17a expression in CD4‐positive gut lymphocytes (Figure S12). During disease progression, AD pathology in the gut is sufficient to induce differentiation and activation of T lymphocytes and myeloid p38α‐MAPK has the opportunity to alter the immune response. Our study suggests that IL‐17a may at least partially mediate the pathogenic role of myeloid p38α‐MAPK in AD pathogenesis. It has been reported that the number of Th17 cells increases in the blood of AD patients (Oberstein et al., 2018). IL‐17a‐expressing T lymphocytes accumulate in the meanings and brain of triple‐transgenic AD mice (3× Tg‐AD; Brigas et al., 2021). It is worthwhile to reanalyze the pathogenic effects of myeloid p38α‐MAPK in AD mice on the basis of IL‐17a deficiency in our future studies.

It should be noted that deficiency of p38α‐MAPK promotes the recruitment of microglia around Aβ deposits in both APP^tg^p38^fl/fl^LysM‐Cre^+/−^ and APP^tg^p38^fl/fl^Cx3Cr1‐Cre^+/−^ mice, possibly favoring Aβ clearance (Hao et al., 2011; Quan et al., 2021). It has also been suggested that microglia clustered around Aβ deposits protect local neurites from damage by forming a physical barrier and condensing Aβ into dense plaques (Condello et al., 2015). Indeed, we observed p38α‐MAPK deficiency in all myeloid cells as well as specifically in microglia protecting neurons in APP‐transgenic mice, albeit with varying efficiency. The mechanism that drives microglia to migrate to Aβ deposits needs to be further identified.

In summary, deficiency of p38α‐MAPK in all myeloid cells, not just microglia, triggers efficient Aβ clearance in the brain and improves cognitive function of APP‐transgenic mice. Together with our previous observations that neuronal deficiency of p38α‐MAPK reduces Aβ and phosphorylated tau proteins in the brains of AD mice (Schnöder et al., 2016, 2020, 2021), our serial studies support that inhibition of p38α‐MAPK is a novel therapeutic option targeting multiple pathogenic processes in AD. As a potential anti‐AD mechanism, deficiency of p38α‐MAPK in peripheral myeloid cells decreases the generation of IL17a‐expressing T lymphocytes, which subsequently activates microglia to internalize Aβ. Further studies on pathophysiological mechanisms associated with IL‐17a‐expressing T lymphocytes may be helpful in optimizing AD therapy with p38α‐MAPK inhibitors.

## 4 EXPERIMENTAL PROCEDURES

### 4.1 Animal models and cross‐breeding

APP/PS1‐double transgenic mice (APP^tg^) over‐expressing human mutated APP (KM670/671NL) and PS1 (L166P) under Thy‐1 promoters (Radde et al., 2006) were kindly provided by M. Jucker, Hertie Institute for Clinical Brain Research, Tübingen, Germany; p38^fl/fl^ mice with loxP site‐flanked *Mapk14* gene (Nishida et al., 2004) were kindly provided by K. Otsu (Osaka University) though the RIKEN Bioresource Center, RIKEN Tsukuba Institute, Japan; Cx3Cr1‐CreERT2 mice that express a fusion protein of Cre recombinase and an estrogen receptor ligand binding domain under the control of endogenous *Cx3cr1* promoter/enhancer elements (Goldmann et al., 2013) were kindly provided by M. Prinz, University of Freiburg, Germany; and LysM‐Cre knock‐in mice expressing Cre from the endogenous *Lysozyme 2* gene locus (Clausen et al., 1999) were bought from The Jackson Laboratory, Bar Harbor, ME (stock number 004781) and were back‐crossed to C57BL/6J mice for >6 generations. APP‐transgenic mice deficient of p38α‐MAPK specifically in myeloid cells (e.g., microglia, macrophages and neutrophils; APP^tg^p38^fl/fl^LysM‐Cre^+/−^) were established by cross‐breeding APP‐transgenic mice with p38^fl/fl^ and LysM‐Cre mice. To generate AD mice with deficiency of p38α‐MAPK specifically in microglia (APP^tg^p38^fl/fl^Cx3Cr1‐Cre^+/−^), APP‐transgenic mice were cross‐bred with p38^fl/fl^ and Cx3Cr1‐Cre mice and induced for the recombination of *Mapk14* gene by intraperitoneal injection of tamoxifen (100 mg/kg; Sigma‐Aldrich Chemie) in corn oil once a day over 5 days. Our study only used mouse litters containing both APP^tg^p38^fl/fl^LysM (or Cx3Cr1)‐Cre^+/−^ and APP^tg^p38^fl/fl^LysM (or Cx3Cr1)‐Cre^−/−^ of genotypes, so that p38α‐MAPK‐deficient and wildtype APP‐transgenic mice were compared between siblings.

To delete IL‐17a in AD mice, APP‐transgenic mice were cross‐bred with *Il‐17a* knockout mice (Nakae et al., 2002), which were kindly provided by Y. Iwakura, Tokyo University of Science, Japan. Moreover, to investigate the location of LysM‐Cre‐expressing cells in the brain, APP‐transgenic mice were cross‐bred with ROSA^mT/mG^ Cre reporter mice (Muzumdar et al., 2007) and LysM‐Cre^+/−^ mice to obtain APP^tg^ROSA^mT/mG^LysM‐Cre^+/−^ of genotype, which express enhanced green fluorescence protein (eGFP) in LysM‐Cre‐expressing cells. To examine whether peripheral myeloid cells migrate into the brain parenchyma, APP^tg^p38^fl/fl^LysM‐Cre^+/−^ were mated to CCR2‐RFP reporter mice (The Jackson Laboratory; stock number 017586), in which the chemokine (C‐C motif) receptor 2 (CCR2) ‐coding sequence has been replaced with monomeric RFP‐encoding sequence (Saederup et al., 2010). To track IL‐17a‐expressing cells in APP‐transgenic mice, APP^tg^ mice were cross‐bred with IL‐17a‐eGFP reporter mice (kindly provided by R. Flavell, Yale University, USA), which express eGFP under the control of mouse *Il‐17a* gene promoter (Esplugues et al., 2011).

Animal breeding, experimental procedure and methods of killing were conducted in accordance with national rules and ARRIVE guidelines, and were authorized by Landesamt für Verbraucherschutz, Saarland, Germany (registration numbers: 40/2014, 12/2018 and 34/2019).

### 4.2 Other experimental methods

Detailed descriptions of: (1) behavior tests, (2) analysis of brain pathology using histological, biochemical, and molecular biological approaches, (3) examination of microglia for their inflammatory activation, morphology, and Aβ internalization, and (4) statistical analysis were provided in the supplemental material.

## AUTHOR CONTRIBUTIONS

Y.L. conceptualized and designed the study, acquired funding, conducted experiments, acquired and analyzed data, and wrote the manuscript. Q.L., L.S., W.H., K.L., Y.D., and I.T. conducted experiments, acquired data and analyzed data. M.M. offered an animal facility and supervised animal experiments. K.F. offered a research laboratory and supervised the laboratory work. All authors contributed to the article and approved the submitted version.

## CONFLICT OF INTEREST

The authors declare that they have no conflicts of interest with the contents of this article.

## Supporting information


Appendix S1

## Data Availability

All data generated or analyzed during this study are included in this published article. Raw data are available upon reasonable request.
