# Supplementary material for: p38α‐MAPK‐deficient myeloid cells ameliorate symptoms and pathology of APP‐transgenic Alzheimer's disease mice
Source: Aging Cell. 2022 Jul 31;21(8):e13679. doi: 10.1111/acel.13679 (PMC9381888; doi:10.1111/acel.13679)
Supplement: Supplementary file 1 — Appendix S1 [file ACEL-21-e13679-s001.pdf]

Qinghua Luo, Laura Schnöder, Wenlin Hao, Kathrin Litzenburger, Yann Decker, Inge Tomic, Michael D. Menger, Yang Liu and Klaus Fassbender

SUPPLEMENTAL INFORMATION

Materials and methods

Morris water maze

The Morris water maze test, consisting of a 6-day training phase and a 1-day probe trial, was used to assess the cognitive function of APP^{tg} mice and their non-APP-transgenic (APP^{wt}) littermates as we did previously (Qin et al., 2016). During training phase, latency time, distance, and velocity of mouse swimming to reach the platform were recorded with Ethovision video tracking system (Noldus Information Technology, Wageningen, the Netherlands). During the probe trial, the platform was removed. The latency of first visit of mice to the location of original platform, the frequency of crossing the location, and the time spent in the platform area were measured.

Collection of brain tissue

Animals were euthanized by inhalation of overdose isoflurane. After perfusion with ice-cold PBS, the brain was removed. The left hemisphere was immediately fixed in 4% paraformaldehyde (PFA; Sigma-Aldrich) for immunohistochemistry. A 0.5-µm-thick piece of tissue was sagittally cut from the right hemisphere and homogenized in TRIzol (Thermo Fisher Scientific, Darmstadt, Germany) for RNA isolation. The remainder of the right hemisphere was snap-frozen in liquid nitrogen and stored at -80°C until biochemical analysis.

Positive selection of CD11b or CD4-positive cells

To determine the gene expression and cell surface antigen expression in microglia/brain macrophages, the entire cerebrum from 4- or 9-month-old APP-transgenic mice were carefully dissected and prepared for a single-cell suspension. As described in a previous study (Liu et al.,

2014), CD11b+ cells were selected with MicroBeads-conjugated CD11b antibody (clone M1/70.15.11.5; Miltenyi Biotec B.V. & Co. KG, Bergisch Gladbach, Germany). Lysis buffer was immediately added to CD11b+ cells for isolation of total RNA with RNeasy Plus Mini Kit (Qiagen, Hilden, Germany); alternatively, CD11b+ cells were used for flow cytometric analysis after stained with Alexa647-conjugated rat anti-mouse CD204 antibody (clone 2F8; Bio-Rad Laboratories GmbH, Feldkirchen, Germany), or were detected with Western blot for Aβ after lysed in radioimmunoprecipitation assay buffer (RIPA buffer; 50mM Tris [pH 8.0], 150mM NaCl, 0.1% SDS, 0.5% sodiumdeoxy-cholate, 1% NP-40, and 5mM EDTA) supplemented with protease inhibitor cocktail (Sigma-Aldrich Chemie GmbH). CD11b+ cells were also selected with magnetic beads-conjugated antibodies from the anticoagulated blood of AD mice for the isolation of RNA.

To select CD4+ lymphocytes, single-cell suspensions were prepared from spleens of 4- and 9-month-old $APP^{tg}p38^{fl/fl}LysM\text{-}Cre^{+/-}$ and $APP^{tg}p38^{fl/fl}LysM\text{-}Cre^{-/-}$ littermate mice with Spleen Dissociation Kit (Miltenyi Biotec B.V. & Co. KG). After blocking with 25 µg/ml rat anti-mouse CD16/CD32 antibody (clone 2.4G2; BD Biosciences, Heidelberg, Germany), cells were selected with Dynabeads®-conjugated antibody against mouse CD4 (L3T4) (Thermo Fisher Scientific). Lysis buffer was immediately added to CD4+ cells for isolation of RNA.

Histological analysis

PFA-fixed left hemisphere was embedded in paraffin and serial 40-µm-thick sagittal sections were cut and mounted on glass slides. For each animal, 4 sections with an interval of 10 layers between neighboring sections were examined. Human Aβ was stained with rabbit anti-human Aβ antibody (clone D12B2; Cell Signaling Technology Europe, Frankfurt am Main, Germany) and microglia labeled with rabbit antibody against ionized calcium-binding adapter molecule -1 (Iba-1; Wako Chemicals, Neuss, Germany) or purinergic receptor P2Y (P2RY12; clone S16007D; BioLegend, San Diego, USA), and visualized with VectaStain ABC-AP kit, or VECTOR Blue Alkaline Phosphatase Substrate kit (both from Vector Laboratories, Burlingame,

USA) or fluorescence-conjugated second antibodies. Compacted Aβ in the brain tissue was stained with Congo red (Sigma-Aldrich Chemie GmbH) according to our established protocol (Liu et al., 2014). In the whole hippocampus and cortex, Aβ area was estimated with the *Cavalieri* method, and Iba-1-positive cells were counted with Optical Fractionator on a Zeiss AxioImager.Z2 microscope (Carl Zeiss Microscopy GmbH, Göttingen, Germany) equipped with a Stereo Investigator system (MBF Bioscience, Williston, USA) using our established protocols (Liu et al., 2014).

The relationship between microglia and Aβ deposits was investigated as we did in a previous study (Hao et al., 2011). Serial brain sections were co-stained with Iba-1 antibody for microglia, and methoxy-XO4 (Bio-Techne GmbH, Wiesbaden, Germany) or Congo red for Aβ deposits. Under Zeiss microscopy with 40× objective, Aβ deposits and surrounding microglia were imaged with Z-stack serial scanning from -10 to +10 μm. From each section, ≥ 10 randomly chosen areas were analyzed. The total number (> 200) of Iba-1-positive cells co-localizing with Aβ deposits were counted. The area of Aβ was measured with Image J (https://imagej.nih.gov/ij/) for the adjudgment of microglial cell number.

To locate GFP-expressing cells in $APP^{tg}ROSA^{mT/mG}LysM\text{-}Cre^{+/-}$ reporter mice, brain sections were serially stained with rabbit anti-GFP (Catalog-number: 600-401-215; Rockland Immunochemicals, Limerick, PA, USA), guinea pig anti-Iba-1 (Catalog-number: HS-234004; Synaptic Systems GmbH, Göttingen, Germany) or mouse anti-NeuN (clone A60; Sigma-Aldrich), and mouse anti-human Aβ (clone: 6C3; Cayman Chemical Company, Michigan, USA), and relevant fluorescence-conjugated second antibodies.

Analysis of microglial morphology

For the analysis of microglial morphology, published protocols and Fiji Image J were used (Fernandez-Arjona, Grondona, Granados-Duran, Fernandez-Llebrez, & Lopez-Avalos, 2017; Young & Morrison, 2018). PFA-fixed brain tissues were embedded in Tissue-Tek® O.C.T. Compound (Sakura Finetek Europe B.V., AJ Alphen aan den Rijn, the Netherlands).

The brain was cut in 30-µm sagittal sections. After fluorescent staining with Iba-1 and Aβ antibodies, total 10 Aβ plaques/mouse were randomly selected from the cortex dorsal to hippocampus and imaged under 40× objective with Z-stack scanning with 1 µm of interval. The serial images were Z-projected with maximal intensity, 8-bit grayscale transformed, Unsharp-Mask filter and despeckle-treated, and binarized to obtain a black and white image. The cells with complete nucleus and branches and without overlapping with neighboring cells were chosen for analysis. The single-pixel background noise was eliminated and the gaps along processes were filled under the view of the original image of the cell. The processed image was skeletonized and analyzed with the plugin Analyze Skeleton (2D/3D) (http://imagej.net/AnalyzeSkeleton) for the total number of primary branches, length of all branches, and the number of branch endpoints of each microglia. The whole analysis was done blinded to genotypes.

The Sholl analysis was performed using the Fiji Image J plugin Simple Neurite Tracer (https://imagej.net/Simple_Neurite_Tracer) and published protocols (Longair, Baker, & Armstrong, 2011; Tavares et al., 2017), by counting the number of microglial branches at a particular distance from the soma.

Western blot analysis

Frozen brain tissues were homogenized on ice in RIPA buffer supplemented with protease inhibitor cocktail and phosphatase inhibitors (50nM okadaic acid, 5mM sodium pyrophosphate, and 50mM NaF; Sigma-Aldrich). The protein levels of synaptic proteins: Munc18-1 protein mammalian homolog (Munc18-1), synaptophysin, synaptosome-associated protein 25 (SNAP-25), and postsynaptic density protein 95 (PSD-95), and β-actin or α-tubulin as a loading control, were detected with quantitative Western blot as we did in previous studies (Quan et al., 2021; Schnöder et al., 2020). For quantification of phosphorylated and total amount of signal transducer and activator of transcription 3 (Stat3), brain homogenates were prepared from 4- and 9-month-old p38α-MAPK deficient and wildtype APP-transgenic mice

and detected with rabbit or mouse monoclonal antibodies against phosphorylated and total Stat3, and β-actin (clone D3A7, 124H6 and 13E5, respectively; all antibodies were bought from Cell Signaling Technology).

Brain homogenates and Aβ ELISA and Western blot analysis

The frozen brain hemispheres were homogenized and extracted serially in TBS, TBS plus 1% Triton X-100 (TBS-T), guanidine buffer (5 M guanidine HCl/50 mM Tris, pH 8.0) as described in our previous study (Liu et al., 2014). Aβ concentrations in three separate fractions of brain homogenates were determined by Aβ42 and Aβ40 ELISA kits (both from Thermo Fisher Scientific). Results were normalized on the basis of the sample's protein concentration.

For detection of Aβ oligomers with our established method (Schnöder et al., 2016), the brain was homogenized in RIPA buffer. The protein was separated by 10 - 20% pre-casted Tris-Tricine gels (Anamed Elektrophorese GmbH, Groß-Bieberau/Rodau, Germany). Anti-human Aβ mouse monoclonal antibody (clone W0-2; Merck Chemicals GmbH, Darmstadt, Germany) and rabbit anti-β-actin monoclonal antibody (clone 13E5; Cell Signaling Technology) were used for Western blot.

Quantitative PCR for analysis of gene transcripts

Total RNA was isolated from mouse brains with TRIzol or from selected cells with RNeasy Plus Mini Kit (Qiagen) and reverse-transcribed. Gene transcripts were quantified with established protocols (Liu et al., 2014; Quan et al., 2021) and Taqman gene expression assays of mouse *Tumor necrosis factor* (*Tnf-α*), *Interleukin-1β* (*Il-1β*), *Inducible nitric oxide synthase* (*Inos*), *Chemokine (C–C motif) ligand 2* (*Ccl-2*), *Il-10*, *Arginase 1* (*Arg1*), *Chitinase-like 3* (*Chi3l3*), *Mannose receptor C type 1* (*Mrc1*), *Apoe*, *Trem2*, *P2ry12*, *Cx3cr1*, *Lpl*, *Clec7a*, *Itgax*, *Scavenger receptor A (Sr-a)*, *Cd36*, *Rage* and *Gapdh* (Thermo Fisher Scientific). The transcription of *Mapk14* genes in CD11b-positive cells was determined using the SYBR green binding technique with the following primers: 5'- CCCGAACGATACCAGAACCT -3' and 5'-CTTCAGCAGACGCAACTCTC -3'.

Statistical analysis

Data were presented as mean ± *SEM*. For multiple comparisons, we used one-way or two-way ANOVA followed by Bonferroni, Tukey, or Dunnett T3 *post hoc* test (dependent on the result of Levene's test to determine the equality of variances). Two independent-samples Students *t*-test was used to compare means for two groups of cases. All statistical analyses were performed with GraphPad Prism 8 version 8.0.2. for Windows (GraphPad Software, San Diego, USA). Statistical significance was set at $p < 0.05$.

References

Fernandez-Arjona, M. D. M., Grondona, J. M., Granados-Duran, P., Fernandez-Llebrez, P., & Lopez-Avalos, M. D. (2017). Microglia Morphological Categorization in a Rat Model of Neuroinflammation by Hierarchical Cluster and Principal Components Analysis. *Front Cell Neurosci, 11*, 235. doi:10.3389/fncel.2017.00235

Hao, W., Liu, Y., Liu, S., Walter, S., Grimm, M. O., Kiliaan, A. J., . . . Fassbender, K. (2011). Myeloid differentiation factor 88-deficient bone marrow cells improve Alzheimer's disease-related symptoms and pathology. *Brain, 134*(Pt 1), 278-292. doi:10.1093/brain/awq325

Liu, Y., Liu, X., Hao, W., Decker, Y., Schomburg, R., Fulop, L., . . . Fassbender, K. (2014). IKKbeta deficiency in myeloid cells ameliorates Alzheimer's disease-related symptoms and pathology. *J Neurosci, 34*(39), 12982-12999. doi:10.1523/JNEUROSCI.1348-14.2014

Longair, M. H., Baker, D. A., & Armstrong, J. D. (2011). Simple Neurite Tracer: open source software for reconstruction, visualization and analysis of neuronal processes. *Bioinformatics, 27*(17), 2453-2454. doi:10.1093/bioinformatics/btr390

Qin, Y., Liu, Y., Hao, W., Decker, Y., Tomic, I., Menger, M. D., . . . Fassbender, K. (2016). Stimulation of TLR4 Attenuates Alzheimer's Disease-Related Symptoms and Pathology in Tau-Transgenic Mice. *J Immunol, 197*(8), 3281-3292. doi:10.4049/jimmunol.1600873

Quan, W., Luo, Q., Hao, W., Tomic, I., Furihata, T., Schulz-Schaffer, W., . . . Liu, Y. (2021). Haploinsufficiency of microglial MyD88 ameliorates Alzheimer's pathology and vascular disorders in APP/PS1-transgenic mice. *Glia*. doi:10.1002/glia.24007

Schnöder, L., Gasparoni, G., Nordstrom, K., Schottek, A., Tomic, I., Christmann, A., . . . Liu, Y. (2020). Neuronal deficiency of p38alpha-MAPK ameliorates symptoms and pathology of APP or Tau-transgenic Alzheimer's mouse models. *FASEB J*. doi:10.1096/fj.201902731RR

Schnöder, L., Hao, W., Qin, Y., Liu, S., Tomic, I., Liu, X., . . . Liu, Y. (2016). Deficiency of Neuronal p38alpha MAPK Attenuates Amyloid Pathology in Alzheimer Disease Mouse and Cell Models through Facilitating Lysosomal Degradation of BACE1. *J Biol Chem, 291*(5), 2067-2079. doi:10.1074/jbc.M115.695916

Tavares, G., Martins, M., Correia, J. S., Sardinha, V. M., Guerra-Gomes, S., das Neves, S. P., . . . Oliveira, J. F. (2017). Employing an open-source tool to assess astrocyte

tridimensional structure. *Brain Struct Funct, 222*(4), 1989-1999. doi:10.1007/s00429-016-1316-8
Young, K., & Morrison, H. (2018). Quantifying Microglia Morphology from Photomicrographs of Immunohistochemistry Prepared Tissue Using ImageJ. *J Vis Exp*(136). doi:10.3791/57648

SUPPLEMENTARY FIGURES

Figure S1, LysM-Cre excises the floxed *Mapk14* gene in microglia and peripheral myeloid cells.

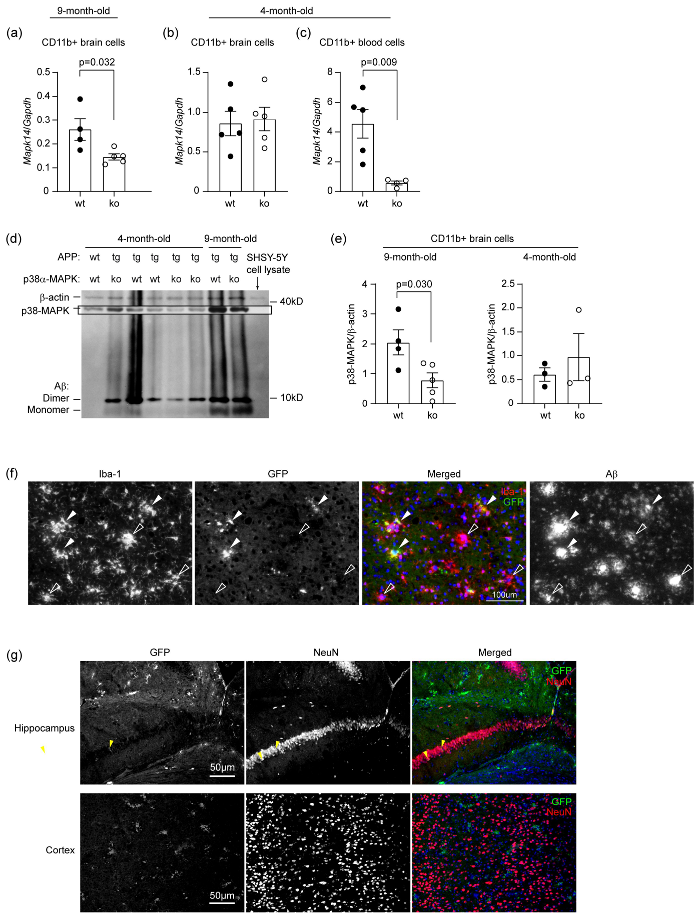

(a-c) CD11b-positive cells were selected from brains and blood of 4 and 9-month-old APP^{tg}p38$^{fl/fl}$LysM-Cre$^{+/-}$ (p38α ko) and APP^{tg}p38$^{fl/fl}$LysM-Cre$^{-/-}$ (p38α wt) mice and measured for transcripts of *Mapk14* gene by real-time PCR. *t* test, $n \geq 4$ per group.
(d,e) To determine the protein level of p38α-MAPK in microglia, the membrane used for Western blot detection of Aβ in the cell lysate of CD11b-positive brain cells was reblotted with an antibody against p38-MAPK (Cat.-No.: 9212; Cell Signaling Technology). LysM-Cre-mediated *Mapk14* recombination significantly reduces p38-MAPK protein in microglia isolated from 9 but not 4-month-old APP-transgenic mice. *t* test, $n \geq 3$ per group. Here representative Western blot images from 4 independent experiments are shown.
(f) Brain sections from 9-month-old APP^{tg}ROSA$^{mT/mG}$LysM-Cre$^{+/-}$ reporter mice were co-stained with fluorescence-conjugated antibodies for GFP, Iba-1 and Aβ. GFP was labelled in Iba-1-positive cells around Aβ deposits (filled arrowheads). Some Aβ deposits were surrounded by Iba-1-positive cells with no or very low GFP expression (open arrowheads).
(g) APP^{tg}p38$^{fl/fl}$LysM-Cre$^{+/-}$ mice were also mated to ROSA$^{mT/mG}$ Cre reporter mice. Paraffin-embedded brain tissues from 6-month-old APP^{tg}p38$^{fl/wt}$LysM-Cre$^{+/-}$ROSA$^{mT/mG}$ mice were co-stained with fluorescence-conjugated antibodies against GFP and neuronal marker, NeuN. We observed that there are very few NeuN-immunoreactive cells in the hippocampus, expressing GFP (marked with arrow heads); and there are no NeuN-positive cells in the cortex, which are positive for GFP staining.

Figure S2, Very few peripheral myeloid cells migrate into the brain of APP-transgenic mice.

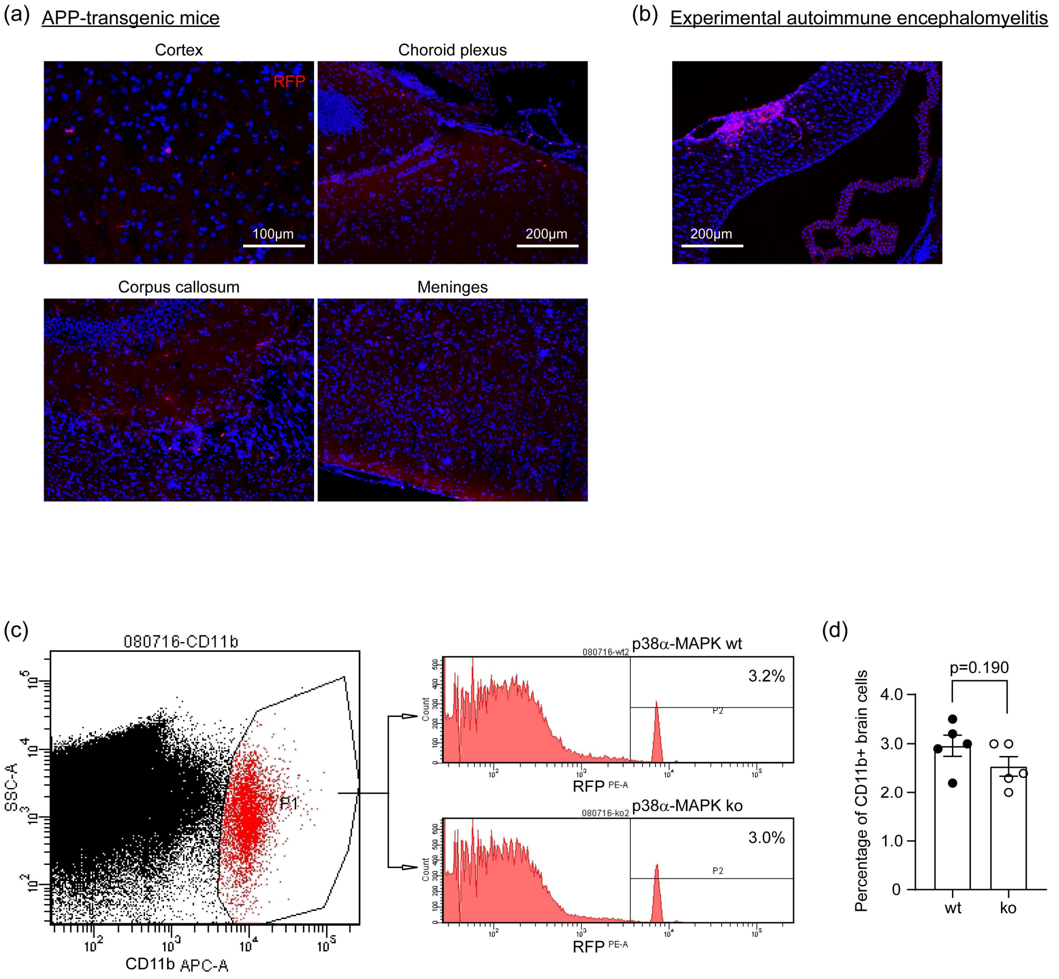

(a) APP^{tg}p38$^{fl/fl}$LysM-Cre$^{+/-}$ mice were cross-bred with CCR2-RFP knock-in mice, which express RFP under the control of *Ccr2* gene promoter. Paraffin-embedded brain tissues from 9-month-old APP^{tg}p38$^{fl/fl}$LysM-Cre$^{+/-}$CCR2$^{RFP/wt}$ mice were stained with RFP antibody (Cat.-No.: 600-401-379; Rockland Immunochemicals, Inc) and Cy3-conjugated anti-rabbit IgG. RFP-immunoreactive cells (in red) with a limited number distribute in different brain regions with a close relationship to blood vessels.

(b) Brain tissues from experimental autoimmune encephalomyelitis models established on CCR2$^{RFP/wt}$ mice were used as a positive control, which shows substantial RFP-positive cells clustering around blood vessels and infiltrating into the brain parenchyma.

(c,d) Single cell suspensions prepared from brains of APP^{tg}p38$^{fl/fl}$LysM-Cre$^{+/-}$CCR2$^{RFP/wt}$ and APP^{tg}p38$^{fl/fl}$LysM-Cre$^{-/-}$CCR2$^{RFP/wt}$ mice were stained with APC-conjugated CD11b antibody and analyzed with flow cytometry for RFP-expressing cells with our established protocol (Liu et al., 2012; https://doi.org/10.1016/j.neurobiolaging.2012.10.015). We observed that there are 2.5% ~ 3.0% of CD11b+ brain cells in APP-transgenic mice potentially derived from peripheral myeloid cells. Deficiency of p38α-MAPK in myeloid cells does not affect the recruitment of peripheral myeloid cells into the brain, as the percentages of CCR2-RFP-positive cells among CD11b-positive brain cells were not significantly different between p38α-MAPK-deficient and wildtype APP-transgenic mice. *t* test, $n = 5$ per group.

Figure S3, Western blot detection of monomeric and dimeric Aβ in the brain homogenate and cell lysate of isolated microglia.

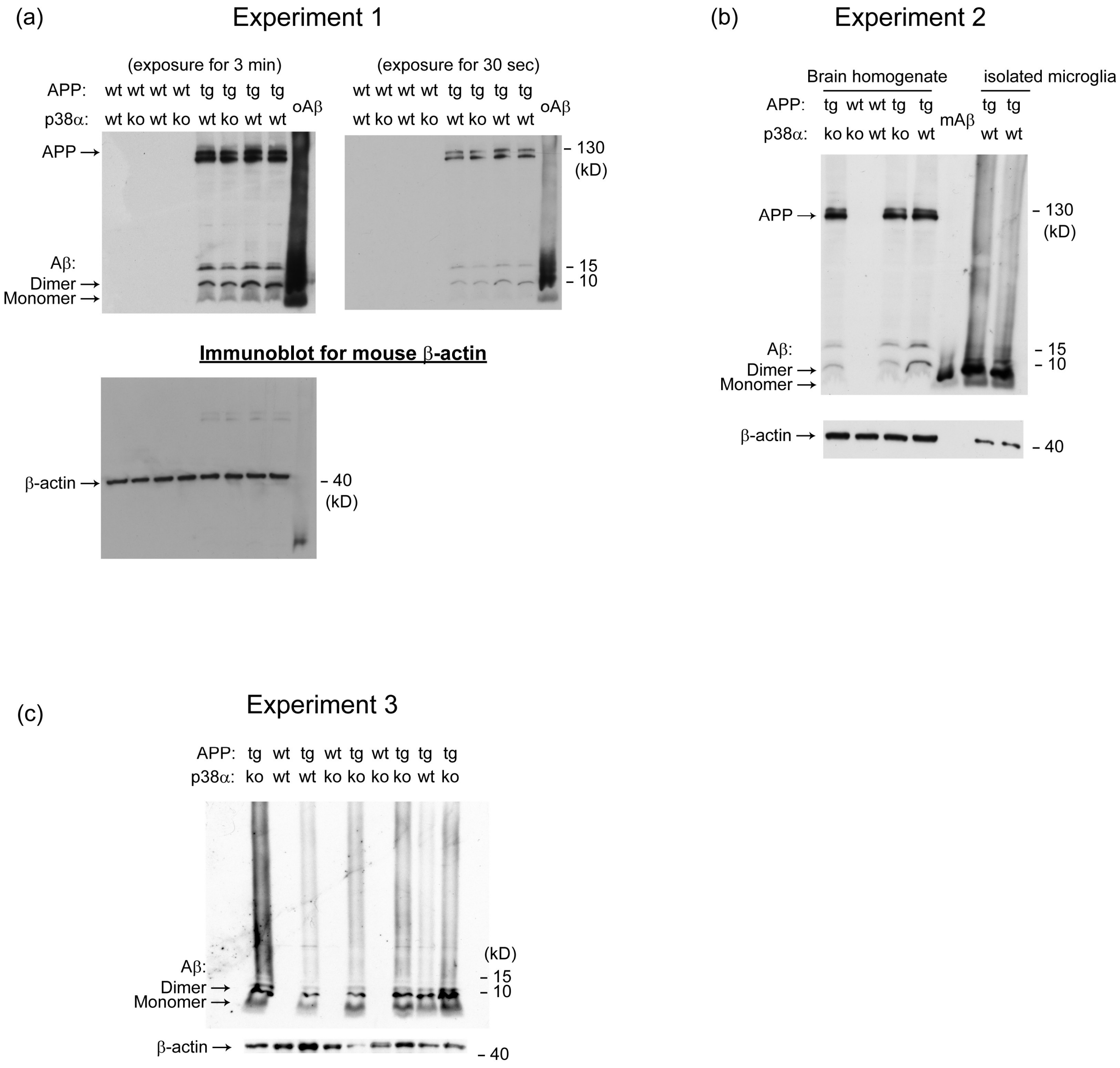

(a-c) Brain tissue from 9-month-old APP-transgenic (tg) and non-transgenic (wt) mice with (ko) and without (wt) deletion of p38α-MAPK in myeloid cells was homogenized in 5 ml/g tissue in RIPA buffer. Microglia were selected with magnetic beads-conjugated CD11b antibodies from single-cell suspensions of brains from the mice described above and lysed directly in Laemmli sample buffer. The proteins were separated by 10-20% pre-casted Tris-Tricine gels (Anamed Elektrophorese GmbH, Groß-Bieberau/Rodau, Germany). For Western blot, anti-human Aβ mouse monoclonal antibody (clone W0-2; Merck Chemicals GmbH) and anti-β-actin rabbit monoclonal antibody (clone 13E5; Cell Signaling Technology) were used. Western blots were visualized via the Plus-ECL method. For each sample, the level of Aβ was calculated as a ratio of target protein/β-actin from that sample. In experiment 1 (a) and experiment 2 (b), synthesized Aβ42 peptides in monomers (mAβ) and oligomers (oAβ) prepared according to the published protocol (Dahlgren et al., 2002; https://doi.org/10.1074/jbc.M201750200) were loaded onto the gel as standard controls. (c) The low part of overall picture of Aβ-immunoblot for microglial cell lysate from experiment 3 was also presented in Figure 4c.

Figure S4, Deficiency of p38α-MAPK in myeloid cells does not change levels of monomeric and dimeric Aβ in the brain of 4-month-old APP-transgenic mice.

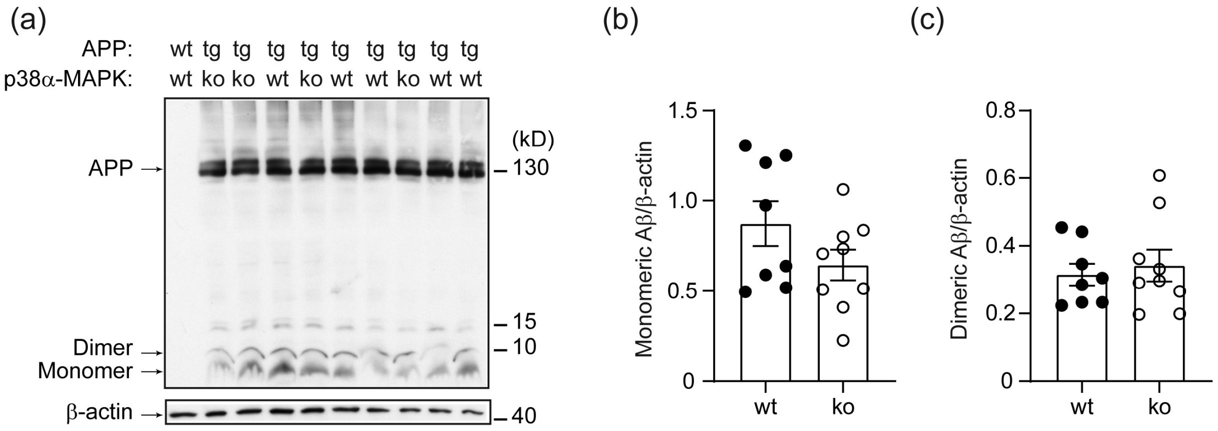

(a-c) Brains of 4-month-old APP-transgenic mice with (p38α ko) and without (p38α wt) deletion of p38α-MAPK in myeloid cells were homogenized in RIPA buffer and detected for Aβ with Western blot. Here, representative Western blot images from 2 independent experiments are shown. Aβ and β-actin immunoblots were performed on the same membrane. *t* test, $n \geq 8$ per group. Data was represented as mean ± *SEM*.

Figure S5, Deficiency of p38α-MAPK in myeloid cells decreases P2RY12-positive microglia in the brain of APP-transgenic mice.

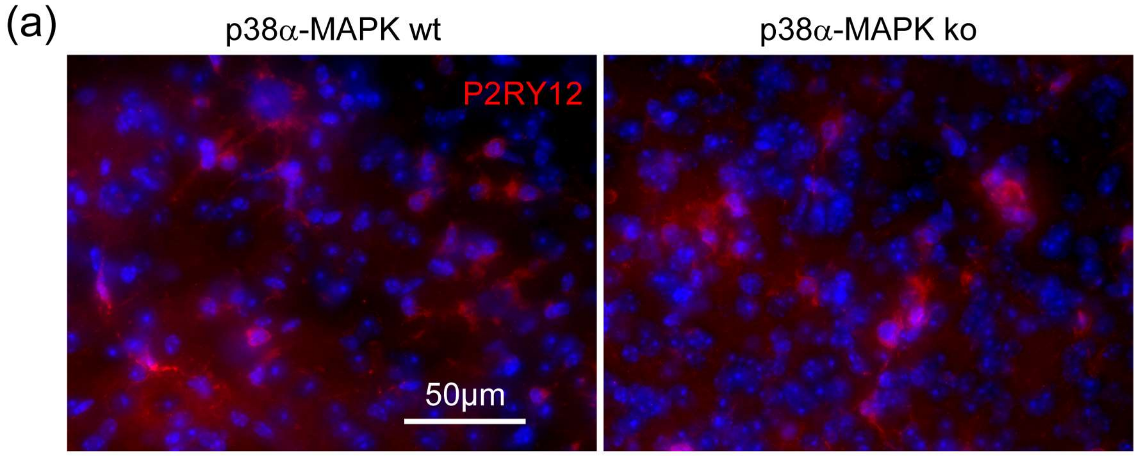

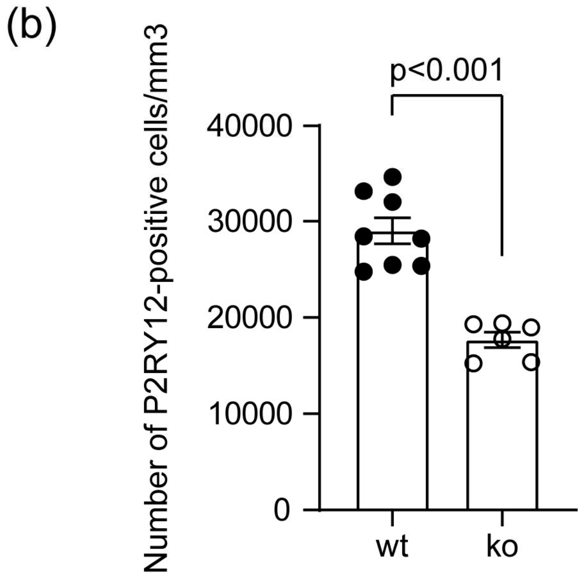

(a, b) Brain sections from 9-month-old APP-transgenic mice with (p38α-ko) and without (p38α-wt) deletion of p38α-MAPK in myeloid cells were stained with rat monoclonal antibody against P2RY12 (clone S16007D; BioLegend) and Cy3-conjugated goat anti-rat IgG (a). (b) Microglia were counted in the hippocampus with the Optical Fractionator stereological probe and adjusted by the volume of analyzed tissues. *t* test, $n \geq 6$ per group.

Figure S6, Deficiency of p38α-MAPK in myeloid cells regulates inflammatory gene transcription in the brain of non-APP-transgenic mice.

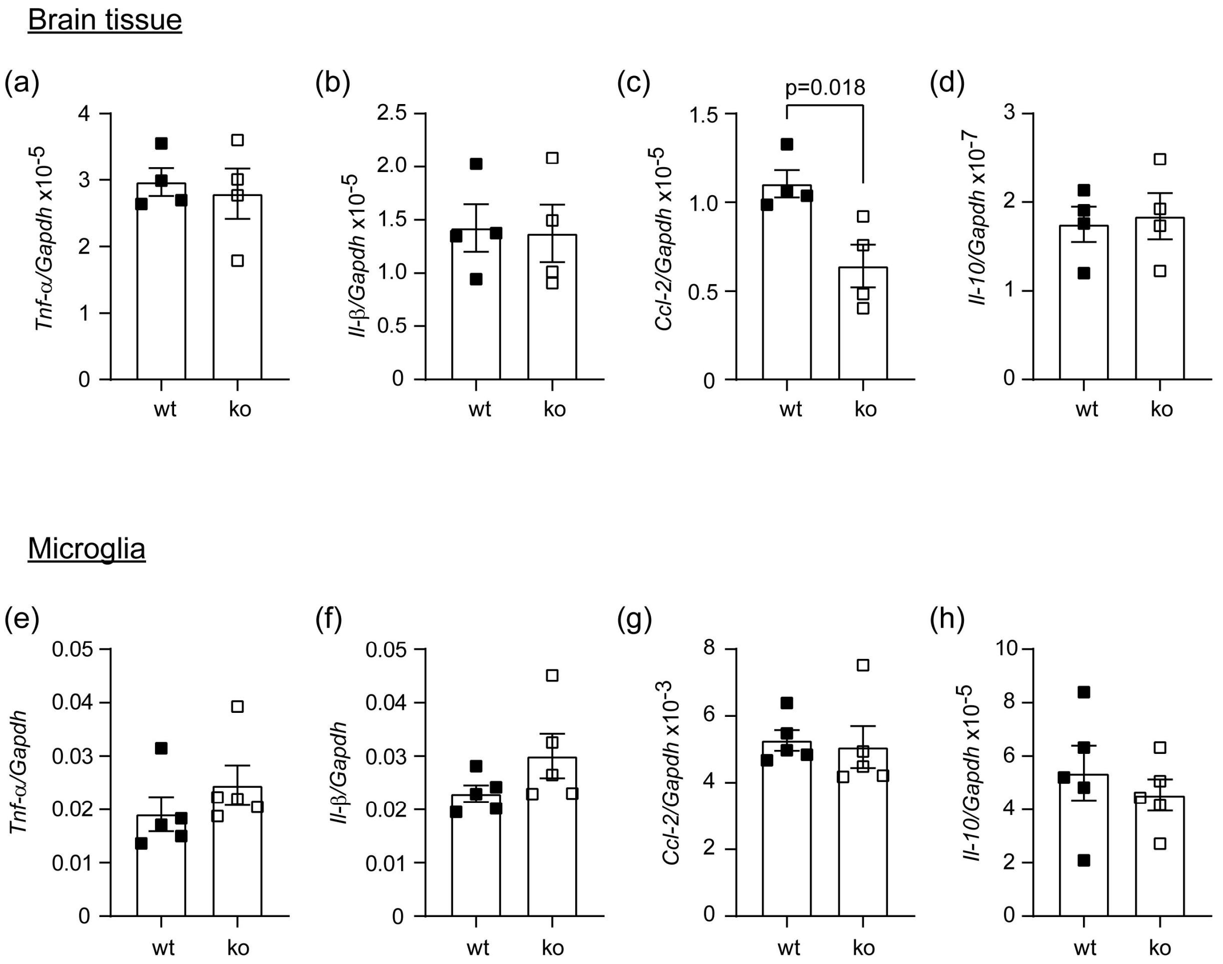

(a-d) Brain tissues from 9-month-old non-APP-transgenic mice with (p38α-ko) and without (p38α-wt) deletion of p38α-MAPK in myeloid cells were measured for inflammatory gene transcripts with real-time RT-PCR. Deficiency of p38α-MAPK down-regulates *Ccl-2* gene transcription in the brain tissue. *t* test, *n* = 4 per group.

(e-h) CD11b-positive cells were selected with magnetic beads-conjugated antibodies from single-cell suspensions of brains from 9-month-old non-APP-transgenic mice. Inflammatory gene transcripts were detected with real-time RT-PCR. Deficiency of p38α-MAPK does not alter the transcription of *Tnf-α*, *Il-1β*, *Ccl-2* and *Il-10* genes in individual microglia. *t* test, *n* = 5 per group.

Figure S7, Deficiency of p38α-MAPK in myeloid cells has limited effects on transcription of inflammatory genes in the brain and microglia of 4-month-old APP-transgenic mice.

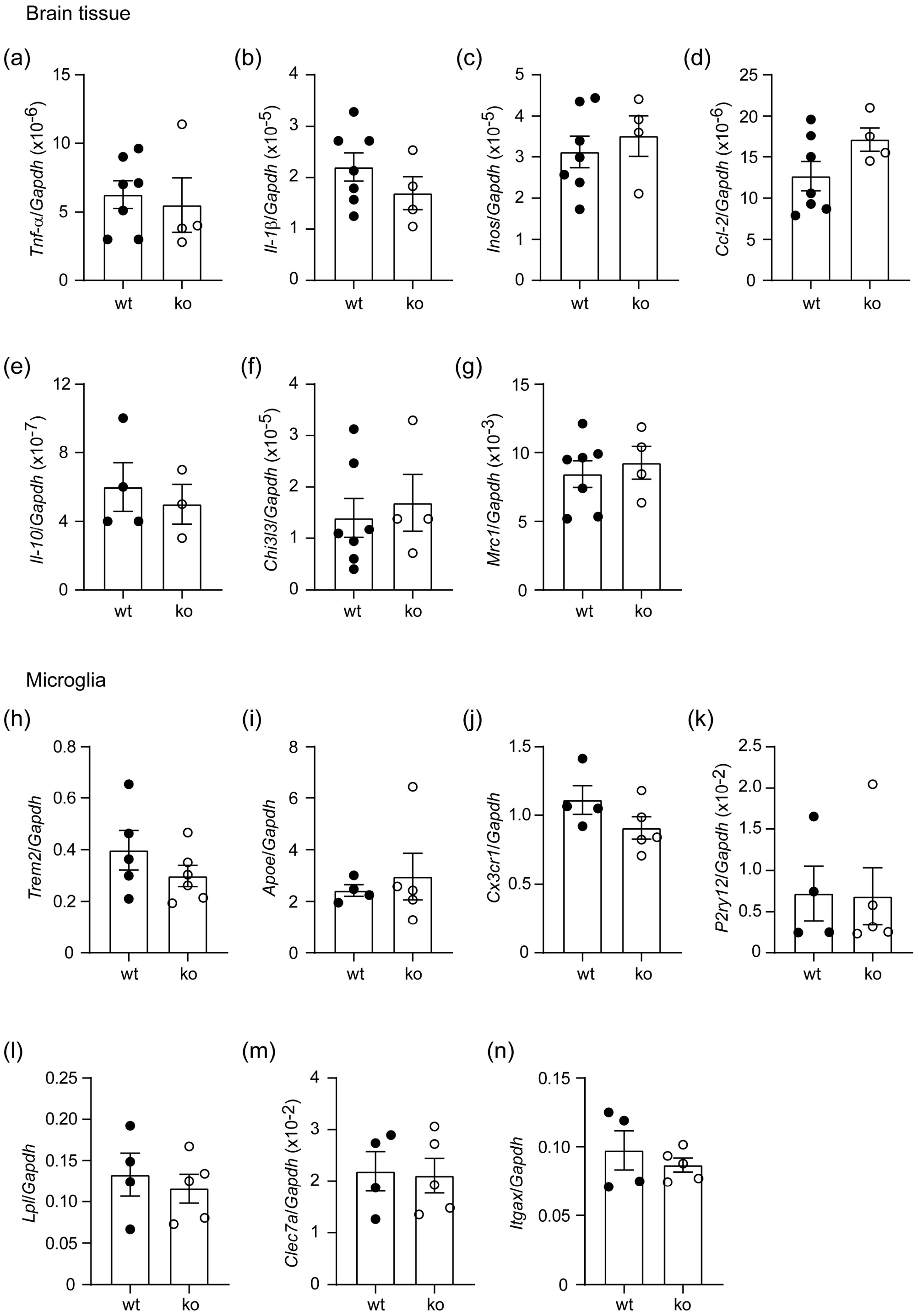

(a-g) Brain tissues from 4-month-old APP-transgenic mice with (ko) and without (wt) deletion of p38α-MAPK in myeloid cells were measured for inflammatory gene transcripts with real-time RT-PCR. *t* test, $n \geq 3$ per group.

(h-n) CD11b-positive cells were selected with magnetic beads-conjugated antibodies from single-cell suspensions of brains from 4-month-old APP-transgenic mice. Inflammatory gene transcripts were detected with real-time RT-PCR. *t* test, $n \geq 4$ per group.

Figure S8, Deficiency of p38α-MAPK in myeloid cells does not affect microglial internalization of Aβ in the brain of 4-month-old APP-transgenic mice.

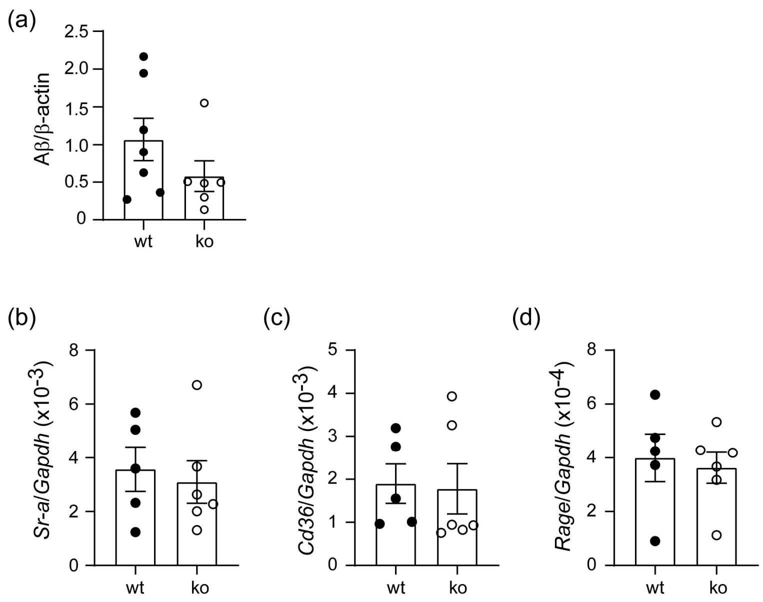

(Figure S1d) and (a) Adult microglia were isolated from 4-month-old APP-transgenic mice with (ko) and without (wt) deletion of p38α-MAPK in myeloid cells, and quantified for intracellular Aβ by Western blot using human Aβ and β-actin antibodies. As a control, no Aβ was detected in the microglia isolated from APP-wild-type mice (Figure S1d). Here, representative Western blot images from 2 independent experiments are shown. Aβ and β-actin immunoblots were performed on the same membrane. *t* test, $n \geq 6$ per group. (b-d) CD11b-positive cells were selected with magnetic beads-conjugated antibodies from single-cell suspensions of brains from 4-month-old APP-transgenic mice. The microglial gene transcription of Aβ internalization-associated receptors, such as SR-A, CD36, and RAGE, was detected with real-time PCR. *t* test, $n \geq 5$ per group. Data was represented as mean ± *SEM*.

Figure S9, Deficiency of p38α-MAPK facilitates Aβ internalization in LPS-primed macrophages.

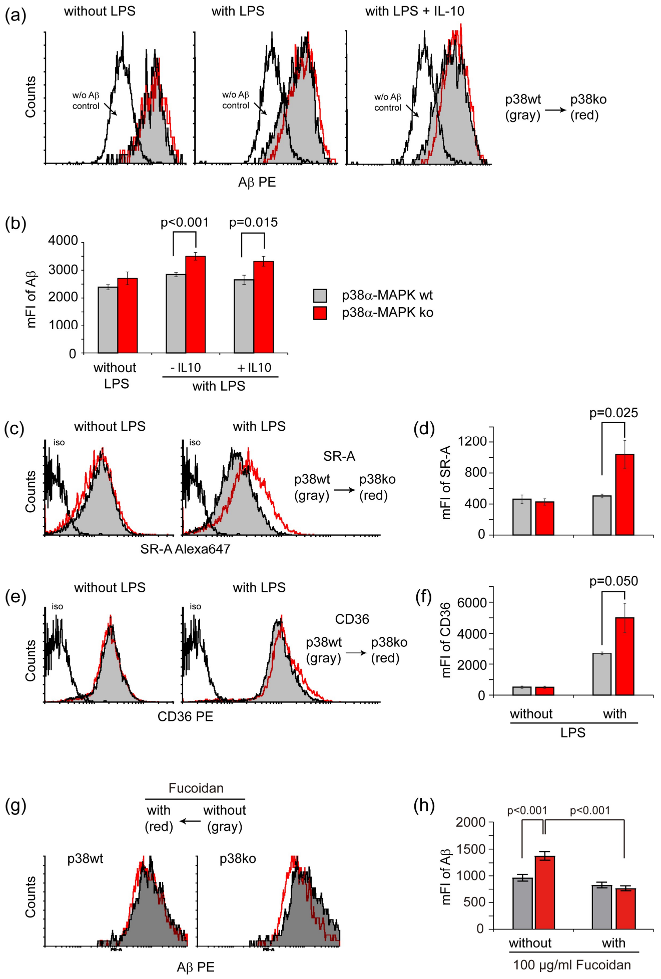

(a,b) Bone marrow-derived macrophages in 24-well plate at a density of 3 × 10^5 cells per well were cultured from $p38^{fl/fl}$LysM-$Cre^{+/-}$ (ko) and $p38^{fl/fl}$LysM-$Cre^{-/-}$ (wt) mice. They were primed with and without 100ng/ml LPS for 48 hours. Some cells were co-treated with recombinant mouse IL-10 at 20ng/ml. Oligomeric aggregates of fluorescent Aβ were prepared by mixing TAMRA-labeled human Aβ42 (Anaspec Inc.) and unlabeled Aβ42 (kindly provided by L. Fülöp, Albert Szent Gyorgyi Medical University, Szeged, Hungary) at a ratio of 1:10 and incubating 100µM mixed peptides in phenol red-free Ham's F-12 for 24 hours, as we did in a previous study (Liu et al., 2014; https://doi.org/10.1523/JNEUROSCI.1348-14.2014). Thereafter, macrophages were fed with 10µM TAMRA-conjugated oligomeric Aβ42 in the presence of LPS with and without IL-10 for 18 hours. The internalization of Aβ was monitored by measuring mean fluorescence intensity (mFI) with flow cytometry. *t* test, $n \geq 6$ per group.

(c-f) Protein levels of SR-A and CD36 on cultured macrophages were detected with flow cytometry after immunofluorescent staining of macrophages with Alexa647-labelled rat anti-mouse SR-A (clone 2F8; Bio-Red), and PE-labeled rat anti-mouse CD36 (clone HM36; Thermo Fisher Scientific), respectively. *t* test, $n \geq 4$ per group.

(g,h) LPS-primed macrophages were pre-treated with 100 µg/ml fucoidan for 1 hour, and then incubated with 10µM TAMRA-conjugated oligomeric Aβ42 in the presence of fucoidan for 18 hours and measured with flow cytometry for mFI of cells. *t* test, $n = 9$ per group.

Figure S10, Haploinsufficiency of Cx3Cr1 affects microglia for neither migration to Aβ deposits nor inflammatory gene transcription in APP-transgenic mice.

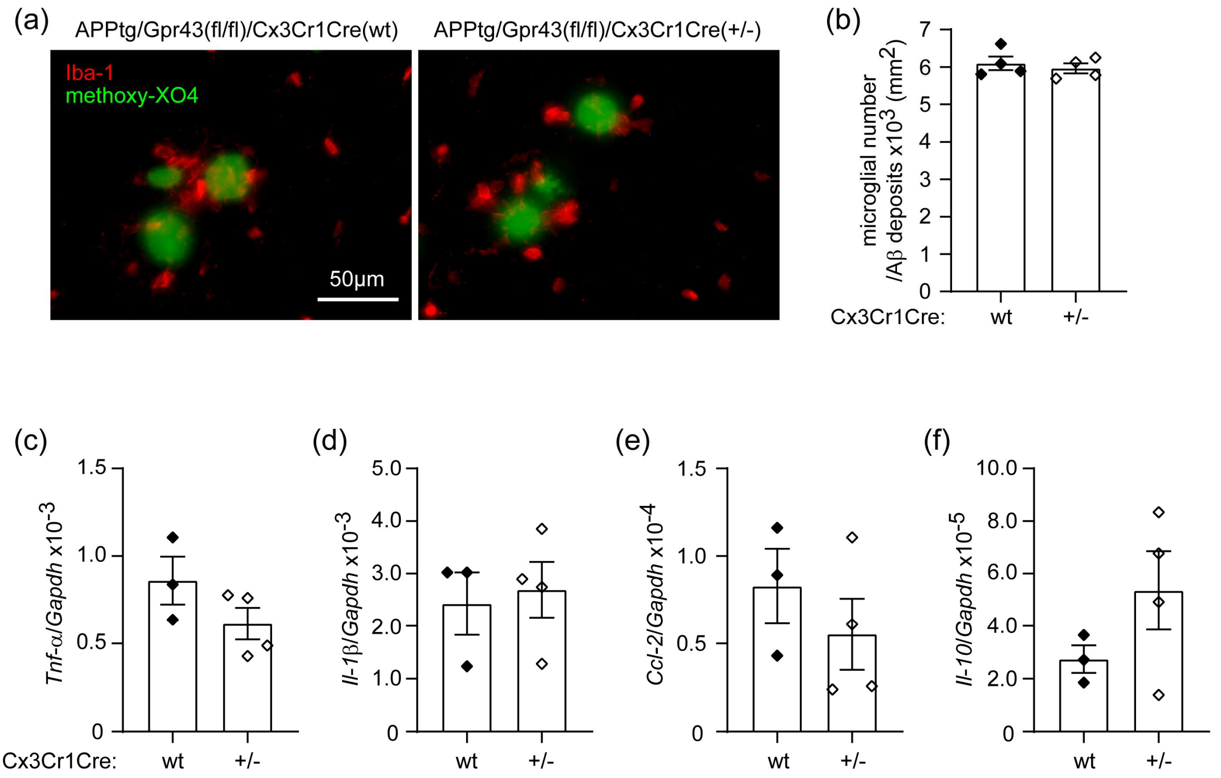

APP-transgenic mice were cross-bred with Cx3Cr1-CreERT2 mice and *Gpr43*-floxed mice (Tang et al., 2015; https://doi.org/10.1038/nm.3779) to obtain mice with $APP^{tg}Gpr43^{fl/fl}Cx3Cr1\text{-}Cre^{+/-}$ and $APP^{tg}Gpr43^{fl/fl}Cx3Cr1\text{-}Cre^{-/-}$ of genotypes. These two groups of littermate mice were injected (*i.p.*) with tamoxifen at 6 months of age and analyzed for AD-associated pathology by 9 months. As GPR43 is not expressed in microglia (Quan et al., 2021; https://doi.org/10.1002/glia.24007), any difference between $APP^{tg}Gpr43^{fl/fl}Cx3Cr1\text{-}Cre^{+/-}$ and $APP^{tg}Gpr43^{fl/fl}Cx3Cr1\text{-}Cre^{-/-}$ mice should come from the haploinsufficiency of Cx3Cr1. In recent studies, we observed that haploinsufficiency of Cx3Cr1 changes neither Aβ load nor inflammatory activation in the brain of 9-month-old APP-transgenic mice (Quan et al., 2021).

(a,b) In this study, we co-stained brain tissues with Cy3-conjugated antibodies against Iba-1 (clone E4O4W; Cell Signaling Technology) and methoxy-XO4 (Bio-Techne GmbH). Iba-1-immunoreactive microglia in contact with methoxy-XO4-stained Aβ were counted and adjusted by the area of Aβ deposits. Haploinsufficiency of Cx3Cr1 does not change microglial recruitment to Aβ deposits. t test, $p > 0.05$, $n = 4$ per group.

(c-f) CD11b-positive cells were selected from single-cell suspensions of brains from 9-month-old $APP^{tg}Gpr43^{fl/fl}Cx3Cr1\text{-}Cre^{+/-}$ and $APP^{tg}Gpr43^{fl/fl}Cx3Cr1\text{-}Cre^{-/-}$ littermate mice. Inflammatory gene transcripts in microglia were detected with real-time RT-PCR. Haploinsufficiency of Cx3Cr1 does not alter the transcription of inflammatory genes, *Tnf-α*, *Il-1β*, *Ccl-2* and *Il-10.* t test, $p > 0.05$, $n \geq 3$ per group.

Figure S11, Deficiency of p38α-MAPK in microglia improves cognitive function of APP-transgenic mice.

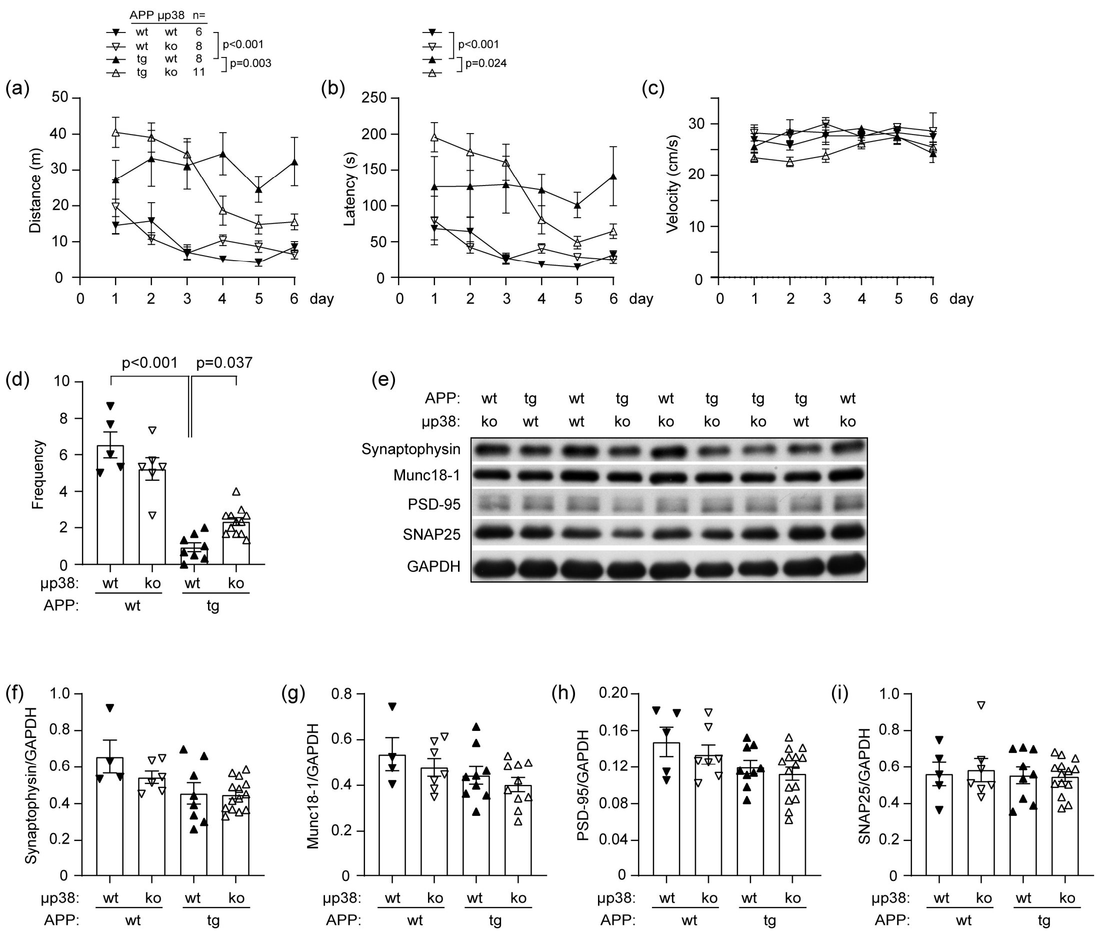

(a-c) 12-month-old APP-transgenic mice (APPtg) and non-APP-transgenic (APPwt) mice with (µp38α ko) and without (µp38α wt) deletion of p38α-MAPK specifically in microglia were analyzed for cognitive function with the Morris water maze test. During the training phase, APPtg mice traveled significantly longer distances (a) and spent more time (b) to reach the escape platform than did their APPwt littermates. Compared to mice with normal expression of p38α-MAPK, deletion of p38α-MAPK in microglia significantly reduced the traveling time and distance of APPtg mice but not of APPwt mice after 3 days of training (a,b). Two-way ANOVA from day 4 to day 6 followed by Bonferroni *post hoc* test, *n* is shown in the figure. (c) Deficiency of microglial p38α-MAPK affected the swimming speed neither of APPtg and APPwt mice, nor for each mouse at different training time points. Two-way ANOVA, $p > 0.05$.

(d) In the probe trial, APPtg mice crossed the region, where the platform was previously located, with significantly less frequency during the total 5-minute experiment than did their APPwt littermates; deletion of p38α-MAPK in microglia partially recovered APP-overexpression-induced memory loss. One-way ANOVA followed by Bonferroni *post hoc* test.

(e-i) Western blotting was used to detect the amount of synaptic structure proteins, Munc18-1, SNAP25, synaptophysin, and PSD-95 in the brain homogenate of 12-month-old APPtg and APPwt mice. Deficiency in microglial p38α-MAPK changed the levels of all these 4 tested proteins neither in APPtg mice, nor in APPwt mice. One-way ANOVA followed by Bonferroni *post hoc* test, $n \geq 9$ per group for APPtg mice and $n \geq 4$ per group for APPwt mice. Here, representative Western blot images from 3 independent experiments are shown.

Figure S12, IL-17a expression is up-regulated in CD4+ lymphocytes of APP-transgenic mice.

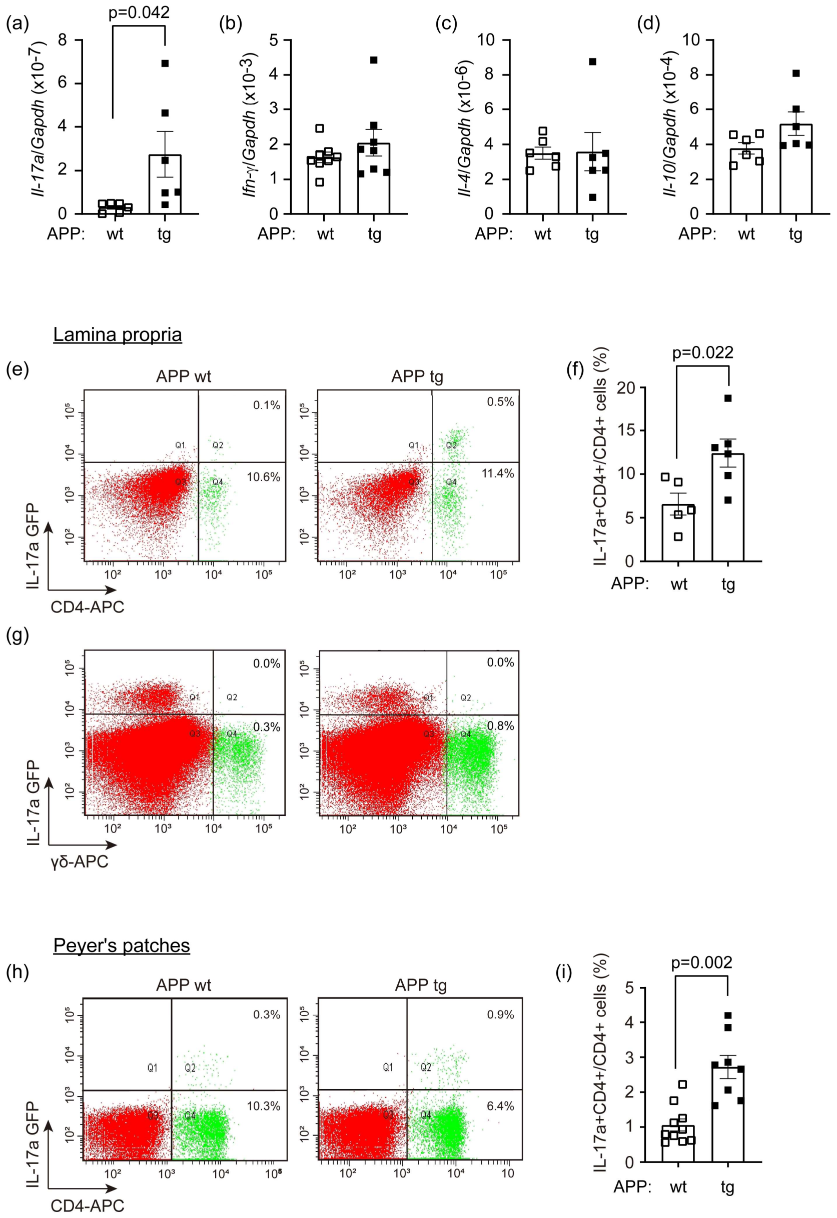

(a-d) CD4-positive splenocytes were selected with magnetic beads-conjugated antibodies from 6-month-old APP-transgenic (tg) and wildtype (wt) littermate mice and detected using real-time PCR for transcription of T lymphocyte marker genes. The transcription of *Il-17a*, but not *Ifn-γ*, *Il-4* and *Il-10* genes, is up-regulated in CD4+ splenocytes of APP-transgenic mice, compared with APP-wildtype littermates. *t* test, $n \geq 6$ per group.
(e-i) APP-transgenic mice were further mated with IL-17a-eGFP reporter mice (Esplugues et al., 2011; https://doi.org/10.1038/nature10228). Single cell suspension was prepared from both lamina propria and Peyer's patches of 6-month-old APP-tg and wt littermate mice using a published protocol (Couter and Surana, 2016; https://doi.org/10.3791/54114), and analyzed using flow cytometry after staining with APC-conjugated rat monoclonal antibody against CD4 (clone: GK1.5) or Armenian hamster monoclonal antibody against γδ T-cell receptor complex (clone: eBioGL3) (i, k, l). IL-17a expression is up-regulated in CD4+ lymphocytes in APPtg mice compared with APPwt littermate controls (f, i). *t* test, $n \geq 5$ per group. GFP was absent in γδ T cells from lamina propria (g) and there were no γδ T cells in Peyer's patches.
